# Comparative Evaluation of Machine Learning Algorithms for Fault Diagnosis in Automotive Press Lines

**DOI:** 10.3390/s26165058

**Published:** 2026-08-09

**Authors:** Ahmet Erdem Oner, Meral Bayraktar

**Affiliations:** 1 TOYOTETSU Automotive Parts and Trading Joint Stock Company, 41420 Kocaeli, Türkiye; onera@toyotetsu.com.tr; 2 Mechanical Engineering Department, Yildiz Technical University, 34349 Istanbul, Türkiye

**Keywords:** machine learning, condition monitoring, fault diagnosis, transfer press, gears

## Abstract

**Highlights:**

- A machine learning-based condition monitoring framework was developed and validated using year-long data collected from industrial automotive transfer presses.
- Random Forest outperformed five competing classifiers, achieving 95% diagnostic accuracy with high precision (97%) and low false-positive rates under real production conditions.
- Synchronized vibration and encoder data enabled angular-domain localization of individual damaged gear teeth, allowing component-level fault identification.
- The proposed methodology provides a practical and scalable solution for predictive maintenance of transfer press gearboxes operating under non-stationary industrial conditions.

**What are the main findings?**

- A data-driven fault diagnosis framework using multimodal sensor inputs achieved high-fidelity gear fault detection, with Random Forest outperforming all models (95% accuracy), followed by k-NN (86%).
- By synchronizing the diagnostic network directly with the encoder, this framework achieved a level of resolution sharp enough to pinpoint component-level degradation at the tooth-26 domain under actual industrial operations.

**What are the implications of the main findings?**

- Our findings validate that deploying nonlinear, ensemble-based learning models provides a highly dependable barrier against false alarms and overlooked failures within intricate production settings.
- This strategy lays down a viable foundation for next-generation, self-governing maintenance pipelines, easing the transition into continuous monitoring and future remaining useful life (RUL) forecasting.

**Abstract:**

Minimizing unplanned downtime is critical for maintaining productivity in modern manufacturing. While combining sensor networks with machine learning provides a practical way to detect mechanical failures early, conventional data-driven diagnostics often fail during highly transient stamping operations. This failure stems from severe spectral smearing and signal distortions caused by fluctuating process loads and variable operating speeds. To address these limitations, we present a field-tested fault diagnosis (FD) framework deployed in an active automotive components plant. Over a twelve-month observation period, we collected raw vibration and process data from two operational transfer presses, building a comparative dataset that captures both localized gear damage and healthy baseline dynamics. After preprocessing the data to isolate signal anomalies, we systematically evaluated the diagnostic performance of six algorithms: SVM, Random Forest, Naive Bayes, k-NN, Decision Trees, and Logistic Regression. By integrating angle-based position data from a high-resolution encoder, the developed framework successfully pinpointed specific defective gear teeth. Ultimately, the Random Forest model outperformed the others, delivering the most robust detection accuracy under real-world factory conditions. These results show that the proposed Condition Monitoring (CM) approach significantly reduces resource waste and prevents costly downtime, offering a practical and scalable asset management model for industrial applications.

## 1. Introduction

Transfer presses are automated machines designed for high-volume production, primarily used in metal-forming and stamping processes. Like other industries, they have been frequently used in both automotive and manufacturing sectors. The increasing need for productivity and accuracy requires employing data-driven and smart maintenance scheduling in transfer presses for rapid, accurate, and reliable anomaly detection (AD) and predictive maintenance (PdM) measures. However, there exists a significant gap between theoretical models and real-world applications, mainly due to the difficulties in maintaining acceptable performance metrics under harsh operational conditions [1].

Likewise, gearboxes are used in a wide range of industrial applications for mechanical power transmission, such as in rotating machinery, including wind turbines and aerospace systems. Their performance is also of great importance, as it is the primary determinant of overall system reliability [2,3]. The important parameters, such as the torque, speed, and rotational direction of a system, are highly dependent on the robustness of the gear teeth since the gearboxes effectively modulate all of them. However, these components are highly susceptible to progressive degradation under the continuous impact loading and extreme conditions, which are typical of industrial operations [4]. Due to their central role in the kinematics chain, even a single gearbox fault might cause total system shutdown, leading to severe secondary failures and important production losses [1]. Timely monitoring of machine health is always important [2].

The aim of applying CM to a gear system is the early prediction of gear failures by detecting, prior to the occurrence of sudden gear failure, degradation due to a fault on the sub-item level, for example, a fault in a gear tooth [5]. It was reported that the maintenance costs account for 15–60% of all manufacturing-related expenses [6] and inadequate maintenance plans compromise a corporation’s entire sustainability model [7]. Traditional maintenance approaches, such as run-to-failure and preventive maintenance, rely on either reacting to breakdowns or performing maintenance at fixed intervals. In contrast, PdM uses real-time data analysis and ML-based predictive modeling to optimize maintenance schedules and extend equipment lifespan [8]. Compared to existing manual data analysis techniques, the utilization of machine learning (ML) has the potential to perform the function of forecasting and anticipating malfunctions through the creation of algorithms that can detect patterns from data and use that understanding to make accurate predictions or choices. In particular, machine learning algorithms are very good at recognizing anomalies in data, learning from patterns, data analysis, and optimization of maintenance schedules [9]. Due to the exorbitant human effort required to create knowledge-driven models for AD, data-driven techniques such as machine learning (ML) are seeing increasing use in PdM applications [10,11]. The integration of machine learning into fault detection for predictive maintenance is crucial as it facilitates the examination of vast quantities of information to recognize patterns and produce precise forecasts. Machine learning supplements maintenance planning in industries by analyzing extensive datasets pertaining to a production process, detecting malfunctions and anomalies, and enabling proactive preventive maintenance strategies [9]. Through the Internet of Things (IoT), even conventional equipment, which often lacks native sensing technologies, can be incorporated into PdM frameworks. A good example of such an application is the sheet metal forming sector, where transfer presses are of great importance for high-precision and productivity operations [12]. Although there are limited studies on industrial metal stamping, various modeling approaches have been proposed [13].

To position the proposed methodology within the broader landscape of industrial asset management, existing diagnostics can be systematically classified into three dominant learning paradigms:

1. Supervised Machine Learning: Supervised learning relies on paired input-output training samples to map predictive relationships. While the model adjusts its parameters on the training set, it is evaluated on a completely independent test set. This unseen test partition serves as the primary benchmark to measure the model’s true generalization capacity [14].

Hwang et al. [15] developed a generalized deep learning-based fault diagnosis model using transfer learning based on vibration data for four different types of servo presses, achieving an accuracy of over 99% across different press types utilizing networks such as CNN, VGG16, and ResNet50. Such studies have taken the existing traditional vibrational analysis methods to a more advanced stage. The efficacy of torsional vibration analysis in the time-frequency domain for fault detection under variable speed profiles was demonstrated by Chen et al. [16]. However, despite the proliferation of gear fault diagnosis techniques, most of them use limited contact-based vibration analysis [17].

Vrba et al. [18] presented a gearwheel model that classifies a single feature extracted from the error signal of an adaptive filter using the SVM method, achieving an average performance improvement of 9% compared to reference methods. Although such model-based approaches necessitate in-depth procedural expertise, the data-driven methods have provided promising results. Zhou et al. [12] managed to monitor mission-part failures by means of SVM, while fault classification for stamping equipment was successfully detected with Long Short-Term Memory networks. Although the accuracy was up to 99% in the mentioned method, it must be noted that this alone is not a sufficient metric for industrial PdM. Because a “False Negative” (missing failure) is considerably more expensive in high-stakes situations than a “False Positive,” ROC-AUC and F1-scores must be used for thorough validation. Past studies reveal that tree-based algorithms often provide an optimal balance between inference speed and predictive power. The comparisons made for SVM, Naive Bayes, and RF have shown that although SVM can achieve high accuracy [19], the Decision Trees (DTs) and ensemble techniques like Gradient Boosted Trees may offer superior recall and explainability [4,20]. RF handles high-dimensional data and imbalanced datasets effectively, though computational complexity is a drawback [21]. Meanwhile, hybrid approaches, such as combining Adaptive ARIMA with RF, can enhance the forecasting of contamination levels and mechanical wear [22]. A paradigm change toward deep learning-based automation is represented by the move to Intelligent Fault Diagnosis (IFD) [23]. Data imbalance is a recurring issue in IFD since machines function normally for the great majority of their service lives, making incorrect data hard to identify and scarce [24]. Unsupervised learning and anomaly detection, which employ algorithms like One-Class SVM or Isolation Forests, offer promising techniques for detecting deviations from “healthy” baselines in such situations without the need for large failure datasets [25]. Algorithm selection studies show that RFs and DTs excel on small datasets, while k-nearest neighbors (kNNs) suits larger ones [26].

2. Unsupervised and Self-Supervised Anomaly Detection: Unlike supervised frameworks, unsupervised learning operates strictly on unlabeled data to uncover hidden structures, patterns, or clusters without predefined target outputs. Eliminating the need for manual labeling makes this approach highly effective for processing massive, complex datasets. By directly analyzing the intrinsic properties of the input features, unsupervised models provide significant flexibility during data collection and exploratory preprocessing phases [27]. For instance, Wang et al. [28] proposed a Spatial-Channel Collaborative Multi-Scale Graph Interaction Deep Transfer Learning (SCMGIDTL) model for unsupervised rotating machinery fault diagnosis, demonstrating robust domain adaptation without target labels. To optimize unsupervised machine fault diagnosis, the SCMGIDTL framework integrates three key mechanisms: spatial-channel feature refinement for high-quality prototype extraction, multi-scale graph interactions for deep cross-domain feature fusion, and a local-global category constraint loss for robust domain alignment. Driven by the architecture, SCMGIDTL delivers outstanding diagnostic performance.

3. Physics-Guided and Physics-Informed Frameworks: These hybrid approaches integrate physical principles or engineering prior knowledge with data-driven algorithms to improve generalizability. A prominent example includes the application of machine-vision-driven physics-informed neural networks for state detection in pantograph–catenary systems, where physical constraints guide the neural network to ensure physically plausible predictions. To improve pantograph–catenary system detection, the recent study developed VIPINN—a machine-vision-driven, physics-informed neural network. By combining a heat diffusion encoder, Fourier neural operator, and liquid time-constant network under a random loss-weighted optimization strategy, VIPINN extracts multidimensional physical features from event-stream images. Their approach effectively captures complex, non-linear dynamic relationships in both the time and frequency domains [29].

Physics-informed machine learning methods belong to data-driven approaches that are trained not only on observed data, but also by incorporating underlying physical laws. Karimpouli et al. applied Gaussian processes (GP) and physics-informed neural networks (PINN) to solve the 1D seismic wave differential equation—one of the most widely used equations in geosciences.

Overall, their study yielded several key insights: while both GP and PINN can successfully solve the differential equation and predict u(x,t) with minimal error, GP achieved a lower error margin than PINN. Although prediction error increased when noise was introduced to the training data, both methods exhibited notable robustness against random noise. Furthermore, both GP and PINN accurately inverted the wave velocity as an unknown parameter in the equation; however, PINN yielded more precise results in this context. Ultimately, PINN offers greater flexibility for incorporating additional physical constraints, making it better suited for inverting multiple unknown parameters [30].

Physics-based and data-driven approaches exist for fault detection in gears. Current fault diagnosis methodologies generally bifurcate into physics-based models—which mathematically simulate failure modes like cracks, pitting, and wear—and data-driven frameworks that leverage raw vibration sensor streams [31]. A persistent gap in traditional gear diagnostics is the over-reliance on artificial, intentionally induced damage, which fails to replicate the natural, progressive degradation (fatigue, wear, and pitting) seen in actual industrial environments [25,32].

Although a lot of work on condition monitoring and fault diagnosis of fixed-axis gearboxes has been reported in the literature, only a few have found their way to industrial applications. The ability of condition statistical indicators is to provide accurate and precise information about the health of various components at different levels of damage [33].

In the real world, gears usually work under variable and fluctuating working conditions [34]. The importance of gear faults under variable working conditions has been taken into account by means of diagnostic analysis in recent vibrational studies. Investigating the non-stationary signals is very complex, and hence several advanced decomposition methods have been recommended. For example, the Empirical Mode Decomposition (EMD) method was combined with multifractional detrended cross-correlation analysis to isolate fault features across varying conditions [35].

Aiming to lessen the inherent mode mixing limitation of standard EMD, Chen et al. [36] integrated Complementary Ensemble Empirical Mode Decomposition and a correlation analysis algorithm to make the selection of intrinsic mode functions for neural network classification. Furthermore, Intrinsic Time-Scale Decomposition and Singular Value Decomposition methods were used [37], while Local Characteristic-Scale Decomposition for denoising in conjunction with vector mutual information was utilized [36].

To bridge this gap, the structural health monitoring literature has increasingly focused on algorithmic robustness under unconstrained factory conditions. For instance, Cheng et al. [38] recently utilized a knowledge distillation framework to tackle bearing fault identification under severely imbalanced sample spaces, balancing diagnostic accuracy with computational efficiency. While their approach counters data scarcity via algorithmic multi-level knowledge transfer from deep teacher-student architectures, our work addresses the non-linear degradation challenge from a physical standpoint. We capture cross-axis, multi-domain vibration profiles and pair them with dynamic structural boundary shifts, namely temperature fluctuations and forming force drops. Exploring these distinct methodological angles—deep knowledge distillation versus regularized, non-linear ensemble architectures—highlights the diverse pathways available to build reliable, false-alarm-resistant prognostic frameworks for modern production lines.

Our study was systematically conducted in two mutually dependent phases.

Phase 1: Macro-Level Anomaly Detection via Machine Learning: Deviating from laboratory-controlled experiments built on simulated faults, this phase leverages a comprehensive, yearlong vibration dataset captured from industrial presses operating under genuine production stress. The primary goal here is to establish a continuous binary screening protocol: determining whether the gearbox is experiencing structural degradation or remaining healthy. Deploying machine learning at this macro stage bypasses the need for resource-intensive signal deconvolution loops during routine, healthy operations, thereby cutting continuous computational overhead. By benchmarking multiple classifiers in the following sections, our empirical data indicates that the Random Forest framework offers the most dependable diagnostic boundaries for transfer press gearboxes, providing a practical blueprint to eliminate resource waste and unplanned downtime.

Phase 2: Micro-Level Fault Localization via Angular Statistical Mapping: Because macro-level classification alone cannot isolate the specific root cause under highly variable operating parameters, a secondary localized diagnostic layer was developed. A high-speed encoder was integrated directly onto the main gear shaft to track instantaneous angular positioning. This shaft orientation data was captured and logged into the database in dead-synchronization with the corresponding vibration streams. A specialized signal processing pipeline was then constructed in Python 3.12 to ingest these synchronized datasets, translating angular positions into deterministic fault location metrics. Through this software, the discrete vibration values corresponding to each individual gear tooth were extracted, and all teeth were mapped onto a Kurtosis chart, thereby enabling the precise detection of the defective gear teeth. Mechanistically, the statistical tooth-localization software operates on an event-triggered architecture. The algorithm remains completely dormant during routine monitoring, activating only when the Phase 1 machine learning model outputs a “Damaged” (Class 1) classification with high statistical confidence. Once this initial alert is triggered, the Python-based diagnostic script executes as a secondary, deep-dive inspection layer. This subsequent phase shifts the focus from broad detection to isolated diagnostics, answering a highly specific operational question: “Which individual gear tooth or specific angular sector is driving the macro-level anomaly?”

Rather than relying on standard time-domain assumptions, our methodology maps high-frequency vibration streams directly onto precise angular coordinates through a synchronized hardware-software interface. Ultimately, the core contribution of this research extends beyond the computational framework itself; it centers on the direct, physical validation of the diagnosed gear defects, which were visually and mechanically verified under actual factory production conditions.

## 2. Materials and Methods

### 2.1. Experimental Setup

**Figure 1:**
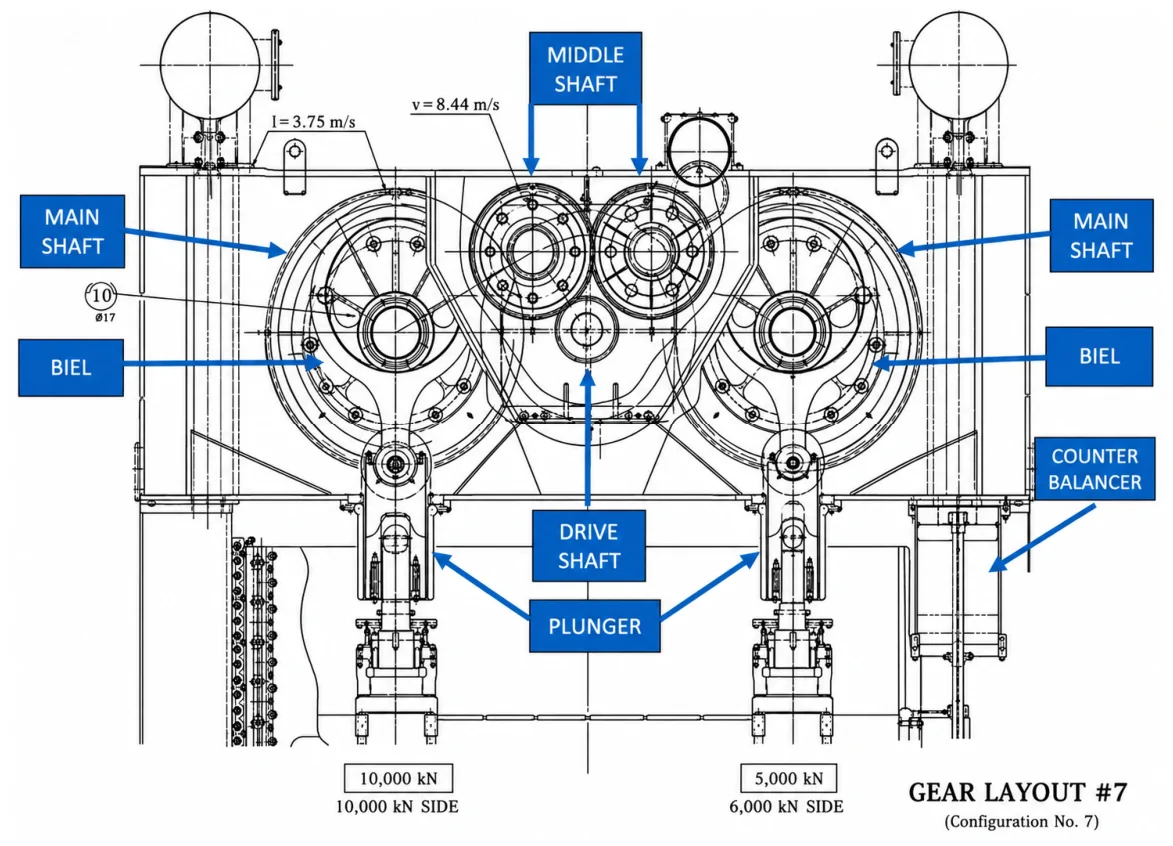
Transfer press gear assembly.

Our twelve-month empirical campaign was conducted directly within an operational automotive supplier facility, capturing the real-world working conditions of a heavy-duty transfer press (KS-Tech Co., Ltd, Toyama, Japan). During the baseline physical inspections of the press transmission system, severe degradation on the main shaft gear was identified (Figure 1).

**Figure 2:**
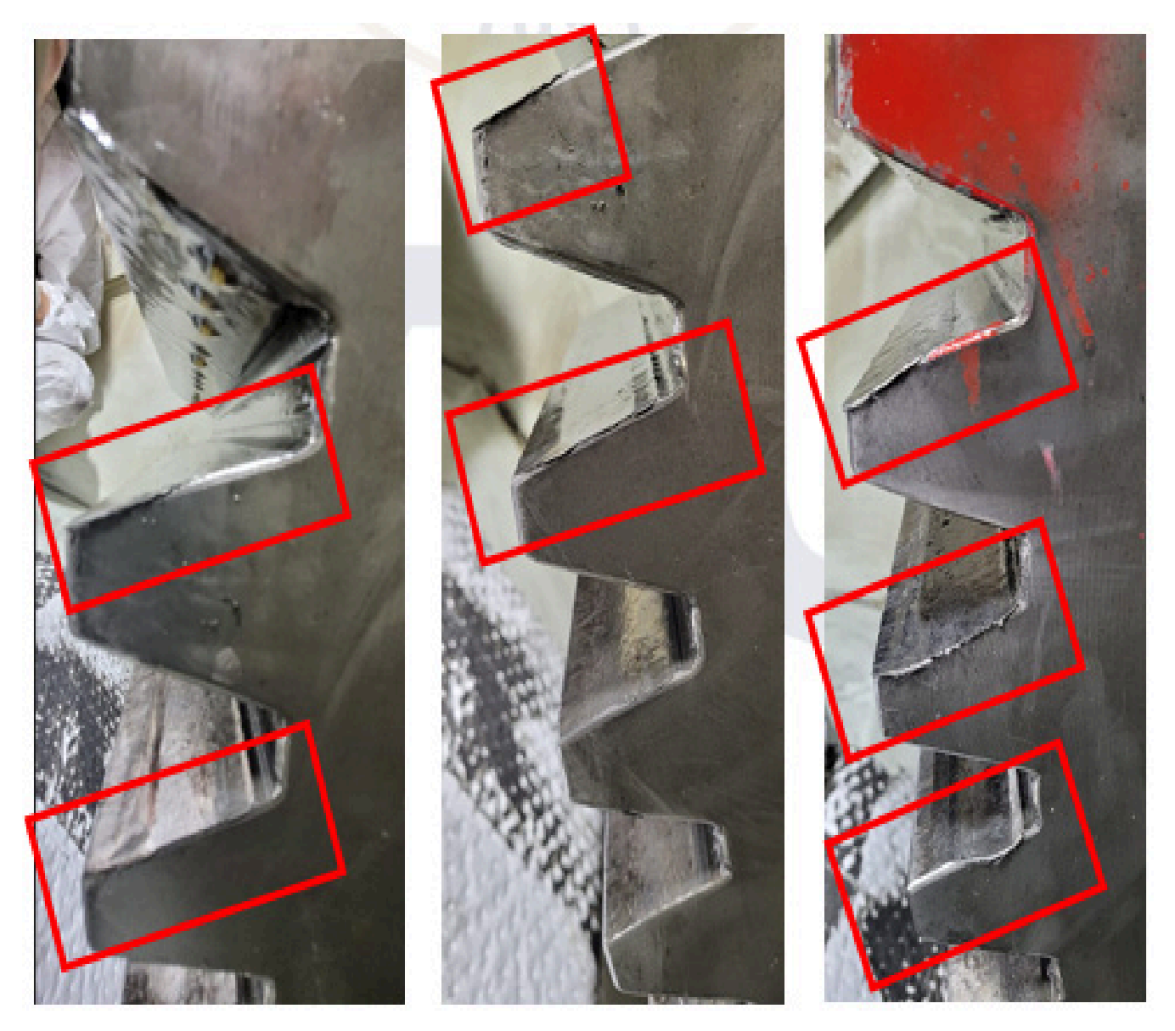
Images of worn gears.

As a direct consequence of this wear, the Bottom Dead Center (BDC) position undergoes spatial deviations. This loss of kinematic precision undermines pressing accuracy (Figure 2), ultimately driving high rejection rates on the production floor.

**Figure 3:**
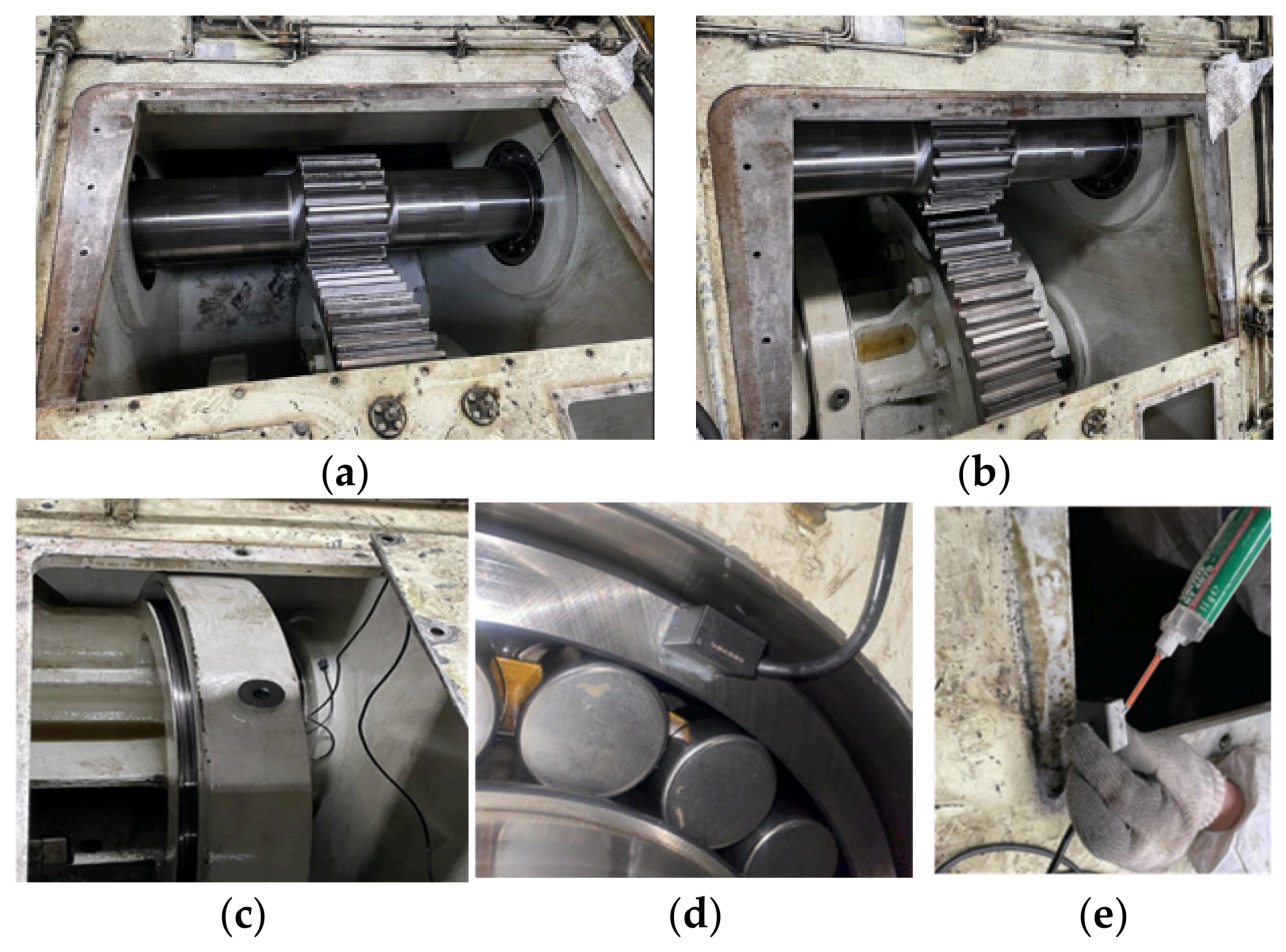
Installation of vibration sensors: (**a**) middle shaft, (**b**) main shaft, (**c**) sensor installation on a sliding bearing, (**d**) sensor installation on a rolling bearing, (**e**) naive use of adhesive in sensor mounting.

We captured the physical footprint of this surface degradation by tracking vibration responses across the affected machinery’s main and middle shafts (Figure 3).

To isolate these fault signatures from background press noise, we deployed the same sensor configuration on an adjacent transfer press equipped with undamaged gears, creating a controlled reference baseline. This parallel monitoring strategy allowed us to assemble synchronous vibration profiles that contrast healthy and faulty states under identical real-world production constraints. For measuring the vibration data, a Sensemore Wired Pro (Sensemore Inc., Istanbul, Turkiye) was employed. This device is a high-precision digital triaxial MEMS accelerometer that also operates as an edge-computing node thanks to its integrated Fast Fourier Transform (FFT) capabilities. Designed for compact industrial integration, the unit measures 26 mm × 24 mm × 10 mm, weighs 15 g, and provides a sampling rate up to 26 kHz. To ensure measurement fidelity, the sensor features a 16-bit ADC resolution, a frequency response range from DC to 6 kHz, and supports a measurement range of ±16 g with a digital sensitivity of 2048 LSB/g.

**Figure 4:**
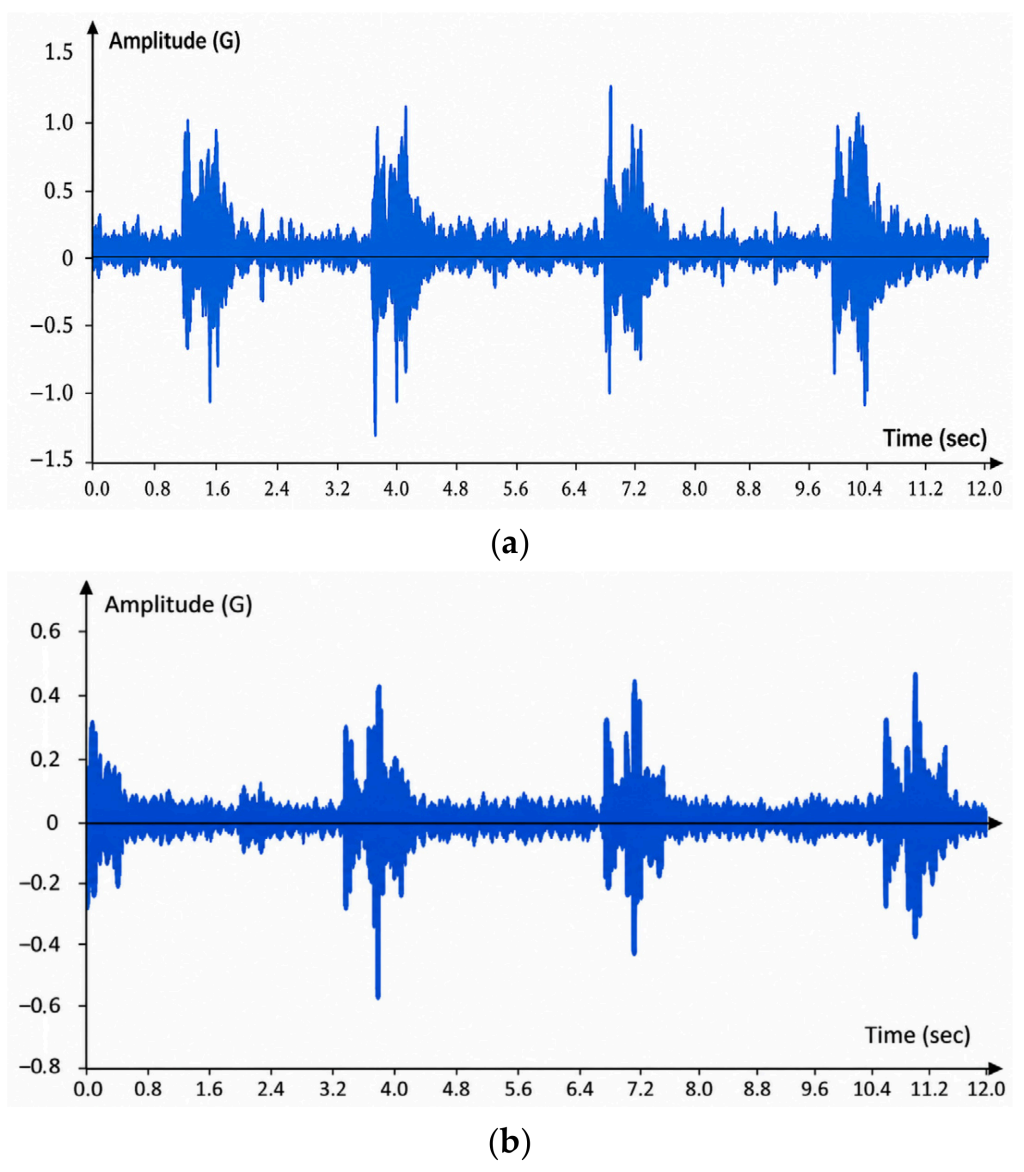
Comparative time-domain waveforms of presses with (**a**) damaged and (**b**) healthy gears.

An involute spur gear with 96 teeth mounted on the main shaft and supported by a sliding bearing makes up the transmission system. The intermediate (middle) shaft features a 20-tooth involute spur gear supported by a spherical roller bearing. Sensor orientation was optimized according to bearing type: radial placement was utilized for the sliding bearings, while axial placement was selected for the rolling-element bearings. Due to these two distinct configurations, the sensor orientations were defined differently during calibration, and the corresponding adjustments for the rolling-element and sliding bearings were configured individually via the sensor software interface. Given a main shaft operational speed of 20 rpm, the rotational speed of the intermediate shaft was determined to be 96 rpm. When the vibration profiles from the same die production were compared between the two presses, the machine with gear degradation showed noticeably larger peak amplitudes (Figure 4). A homogeneous load distribution and the lack of mechanical abnormalities were shown by the healthy gearset’s reduced amplitudes in both time-domain and spectral examinations. On the other hand, gear wear causes impulsive impacts, backlash, or slippage by altering the load-sharing profile throughout the tooth mesh. High-amplitude, non-stationary abnormalities that can be seen in the temporal domain are the manifestation of these phenomena. The raw temporal data were converted into the frequency domain using FFT in order to quantify these abnormalities. A Hanning window was used to reduce spectrum leakage and improve the resolution of the diagnostic peaks.

**Figure 5:**
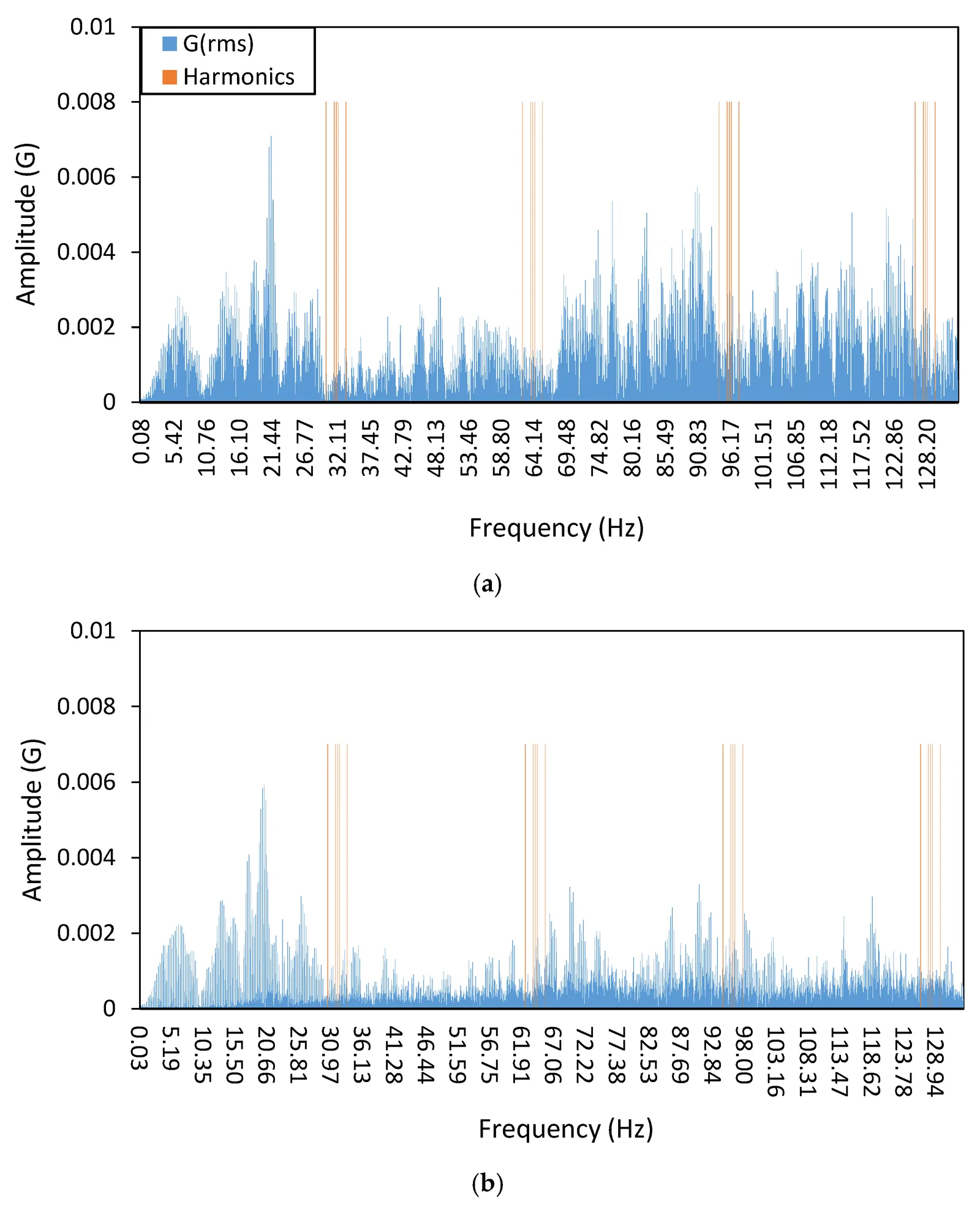
Spectrum diagrams of presses with damaged (**a**) and healthy (**b**) gears.

The Gear Mesh Frequency (f*_GMF_*) is a critical parameter for identifying tooth-related defects in spectral analysis. For the main shaft, the f*_GMF_* was calculated as 32 Hz using the following relationship:

(1) $$f_{GMF}=\frac{N\times n}{60}$$

where *N* and *n* are the number of gear teeth and rotational speed in RPM (Revolution Per Minute), respectively. The sideband frequencies are determined by the rotational frequencies of the respective shafts (see Figure 5). For the main shaft operating at 20 RPM, the modulation frequency is $f_{main}=20/60=0.33\;Hz$, while for the intermediate shaft operating at 96 rpm, the modulation frequency is $f_{middle}=96/60=1.6\;Hz$. These sidebands, which emerge as a result of amplitude and frequency modulation caused by gear defects, are calculated as 32±0.33 →31.67 Hz~32.33 Hz and 32±1.6 →30.4 Hz~33.6 Hz. In the subsequent spectral analysis graphs, the *f_GMF_* and its associated sidebands are highlighted with solid orange lines to facilitate the identification of mechanical anomalies.

Gear tooth degradation characteristically concentrates energy at the GMF and its associated higher-order harmonics. Rather than producing smooth waveforms, these defects introduce sharp, impulsive vibration spikes that significantly elevate amplitude thresholds during spectral analysis. We can trace this energy expansion in both time and frequency domains directly to non-uniform load distributions and recurring mechanical impacts. While a direct comparison of the spectral data reveals that the damaged press generates much higher peak amplitudes than the baseline unit, the resulting spectral profile lacks the clean, symmetrical geometries documented in laboratory literature. The distinct symmetric sidebands that typically signal gear failure are distorted in this industrial environment, indicating that raw diagnostic signals are heavily modulated by background factory noise and process interference.

These limitations prove that traditional frequency-domain screening alone cannot support reliable fault detection in heavy manufacturing settings, which motivated our development of the machine learning pipeline and the synchronized angular-domain statistical software detailed below.

### 2.2. Encoder Synchronization and Angular Mapping Method

Correlating vibration transients with the angular position of the primary gear set required mounting an incremental encoder (1000 PPR) (NSD Corp, Ltd., Aichi, Japan) on the main shaft to deliver high-resolution rotational feedback. Interfacing this mechanical hardware with the digital database was achieved by integrating an OMRON CP1L Programmable Logic Controller (PLC) (OMRON Corp., Kyoto, Japan) and an associated control panel into the architecture, which converted the raw encoder signals into accessible analog values. The full 360° rotation was linearly mapped between 0 and 10 V scales to properly quantify the encoder angle. This 0–10 V analog representation was then digitized through the built-in analog-to-digital converter (ADC) of the OMRON PLC, utilizing a quantization resolution of 4000 steps. Operating under this configuration provides a voltage sensitivity of 2.5 mV/step, which mathematically yields a highly precise angular resolution of 0.09° per step (360°/4000).

**Figure 6:**
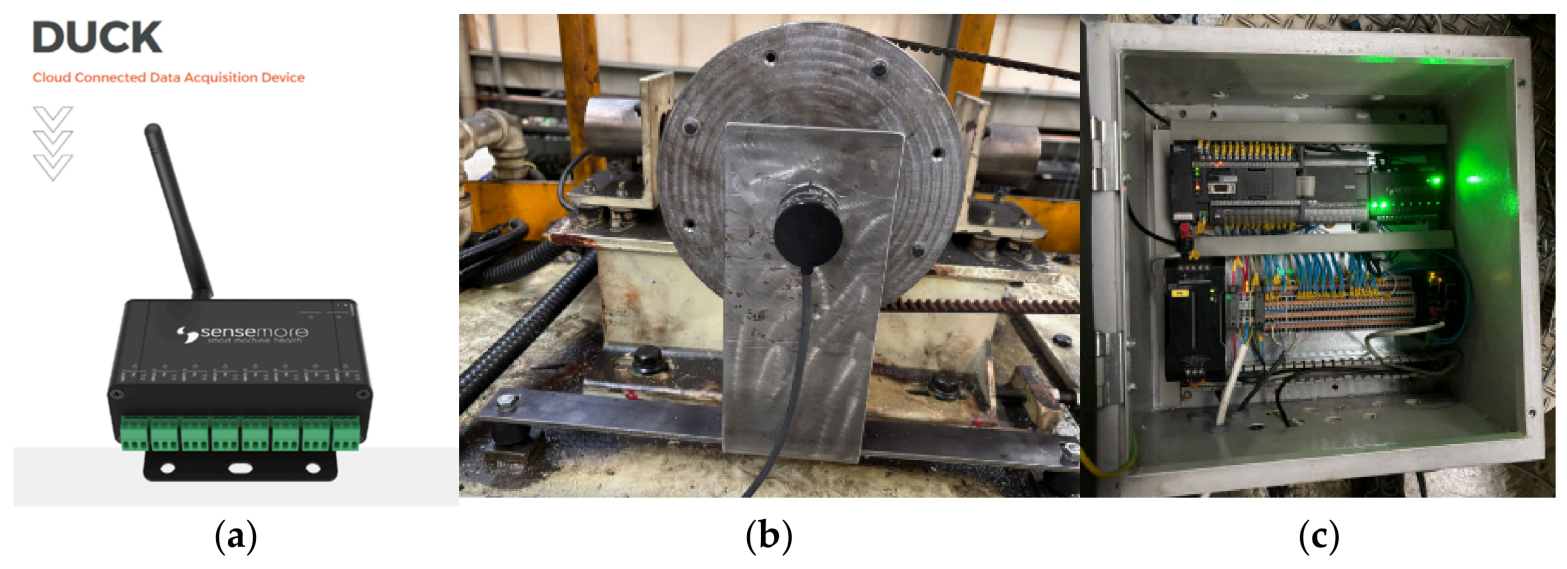
(**a**) Gateway (Duck), (**b**) encoder and (**c**) PLC board.

Finally, feature-level concatenation was handled by a dedicated industrial IoT gateway—specifically the Sensemore “Duck” model—enabling the simultaneous acquisition of vibration signatures and shaft position metrics (Figure 6).

The Duck is a compact data acquisition hardware designed to collect process parameters, including vibration, temperature, and pressure, as well as various analog signals such as mass flow rate, speed, and current. The technical specifications of this acquisition module include 8 channels, a 24-bit resolution, a sampling rate of 4.8 kHz, and data transmission over a 2.4 GHz frequency band (Wi-Fi 802.11 b/g/n), powered by a 12–24 VDC input. Given the 4.8 kHz sampling frequency, this hardware-level data concatenation guarantees deterministic synchronization accuracy of approximately ±0.21 ms (1/4800 Hz) between the geometric position and the dynamic vibration signal.

**Figure 7:**
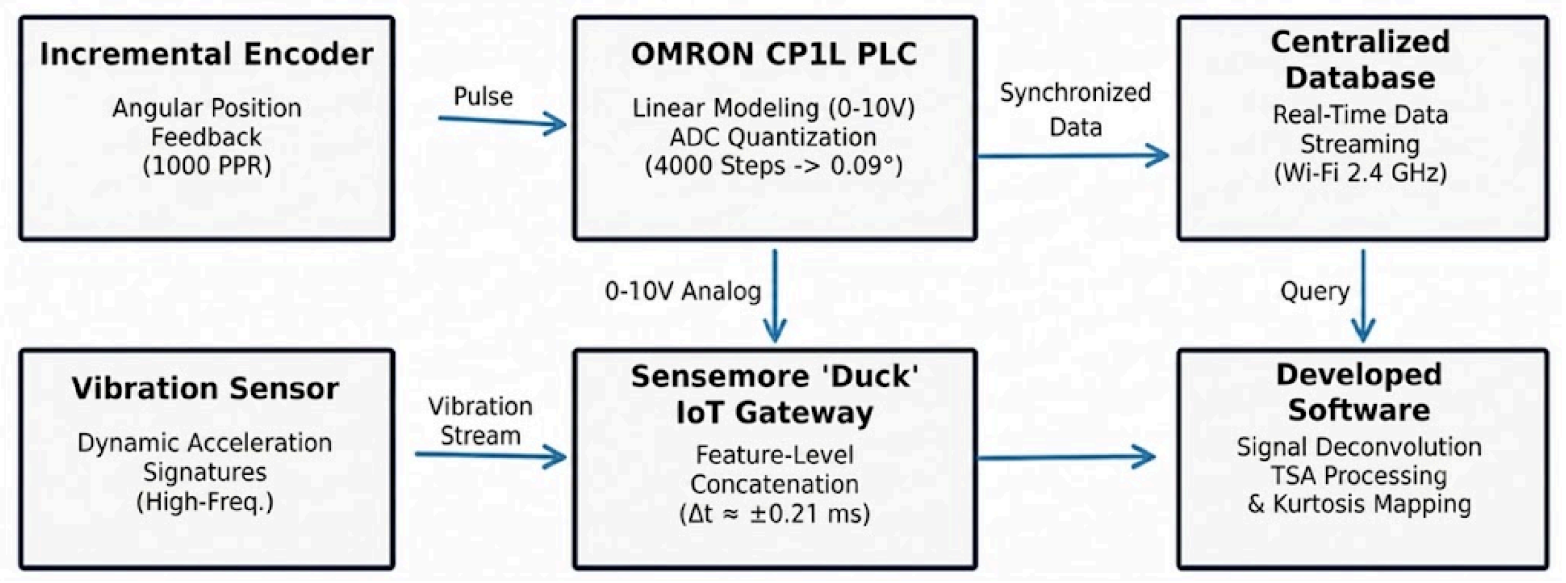
The integrated hardware architecture and systematic data flow designed for synchronized high-frequency vibration and angular position data acquisition.

By synchronizing these high-frequency data streams, the system successfully recorded operational parameters into a centralized database in real-time. We ingested the synchronized vibration and position data into a custom Python-driven diagnostic tool designed for high-resolution tracking. This software utilizes signal deconvolution paired with time-synchronous averaging (TSA), transforming raw high-frequency vibration transients into exact angular coordinates for each gear tooth. Merging the incremental encoder feedback with the vibration envelope allowed the system to extract the distinct kinematic footprint of every single tooth. We selected Kurtosis as our core degradational metric due to its extreme sensitivity to impulsive shocks and spalling, calculating it for each tooth to construct the “Kurtosis Map” shown in Figure 7.

Ultimately, this spatial mapping isolates statistical outliers directly within the meshing cycle. This setup provides the tracking precision needed to isolate micro-level defects on specific gear teeth, maintaining total invariance against operational shaft speed fluctuations instead of just flagging broad, macro-level gearbox wear.

## 3. Machine Learning Framework

### 3.1. Data Collection

**Figure 8:**
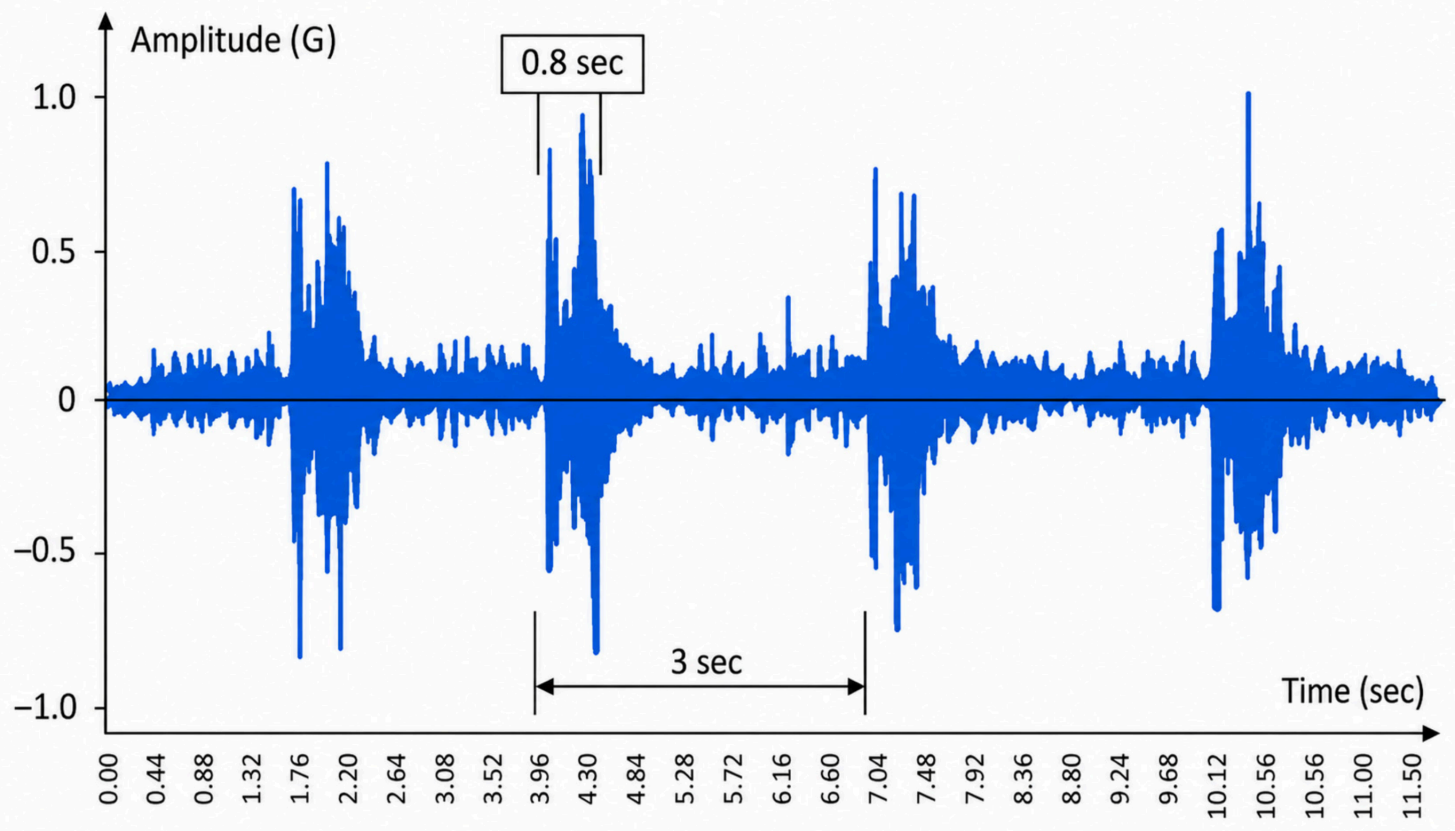
Time domain waveforms of presses.

We established a year-long monitoring campaign from May 2024 to May 2025 to capture a longitudinal dataset from two distinct transfer presses: Press A (operating with active gear degradation) and Press B (running with intact gear sets). This long-term window allowed us to record synchronous vibration, force, and temperature profiles under real-world factory workloads, ensuring the data accounted for seasonal environmental shifts and routine operational variations. Active production cycles yielded a total of 1,110,000 triaxial (X, Y, Z) acceleration data points, mapping the entire 360-degree kinematic rotation of the press drivetrain. We controlled for process-related noise by manufacturing the exact same part on both machines using identical stamping parameters. Sampling occurred in 12 s blocks, from which we isolated 510,000 points from Press A and 600,000 points from Press B. To prevent bias during machine learning model development, we combined these segments sequentially in an alternating pattern (one healthy sample followed by one faulty sample), ensuring a well-mixed training space. We then partitioned the compiled data into a 70% training and 30% testing split. To home in on the critical mechanics of the press stroke, we segmented the raw waveforms around the BDC phase, where maximum mechanical impact occurs. With a 3 s cycle duration and an 835 Hz sampling rate (Figure 8), the impact energy typically peaks and stays elevated for 0.6 s. We therefore extracted data in 0.8 s windows to fully capture the transient dynamics leading into and out of the peak impact, establishing a robust foundation for downstream feature classification.

**Figure 9:**
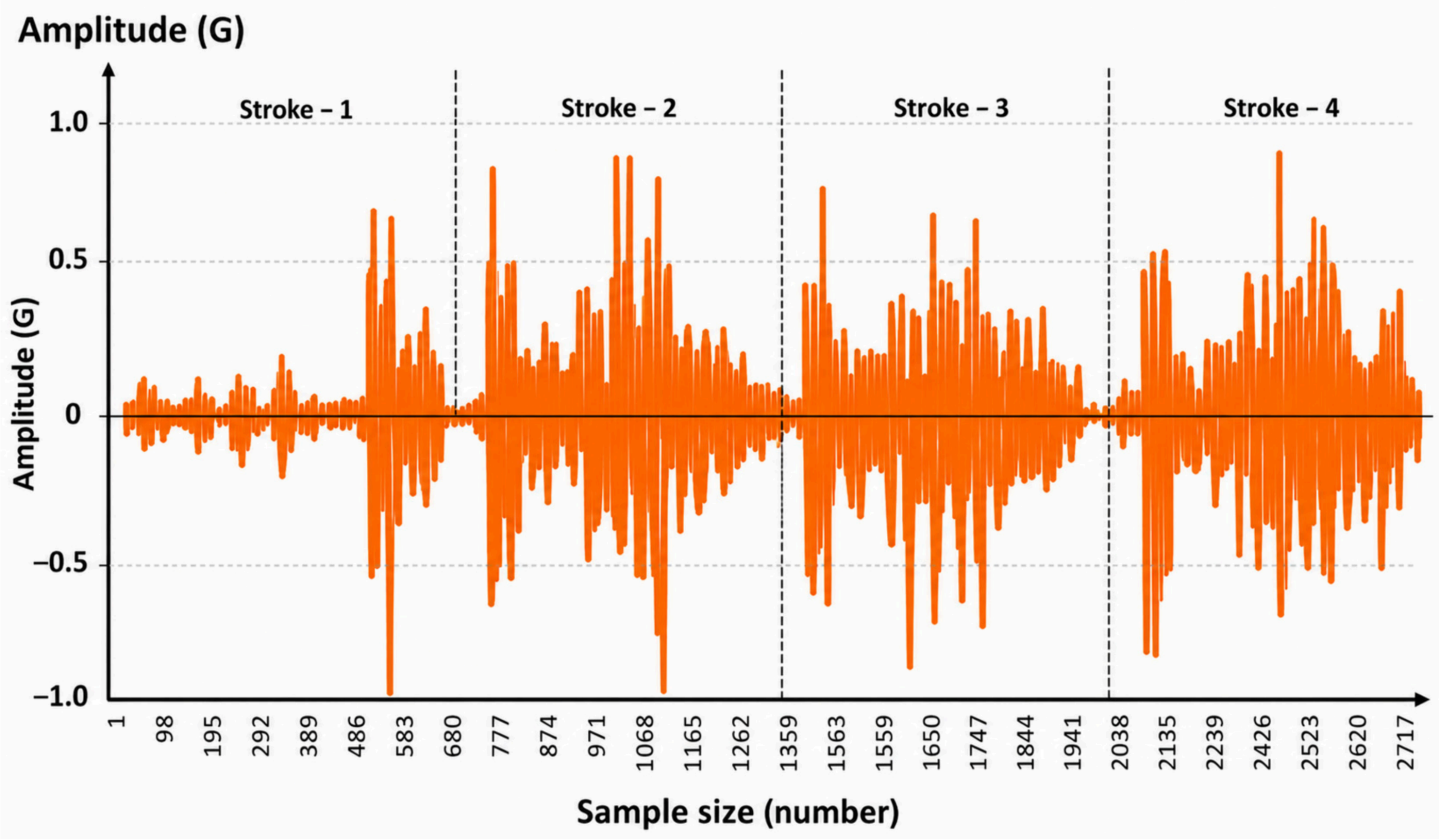
Amplitude–sample size diagram.

By pairing our 835 Hz sampling rate with the 0.8 s window duration, we isolated precisely 668 data points for each discrete impact event while filtering out irrelevant inter-cycle background noise. During the initial reconstruction of these segmented signals, we observed significant baseline irregularities across the early impact sequences. Our technical troubleshooting revealed that these variations stemmed from a synchronization latency, which introduced a 0.5 s delay right at the start of the data acquisition trigger. We resolved this issue and protected the training set from transient artifacts by filtering out the very first impact cycle of each recording session (Figure 9). This processing step ensured that our machine learning classifiers ingested only stable, cyclically consistent operational data.

By applying our targeted filtering and segmentation protocols, we refined the raw dataset from 1,110,000 initial points to a consolidated, high-quality subset of 262,500 data points. We allocated exactly 87,500 samples to each individual axis (X, Y, Z) and serialized them via end-to-end concatenation to safeguard temporal continuity. Our initial capture phase had generated 1,110,000 raw samples over continuous 12 s intervals, spanning roughly four complete strokes of the mechanical press (3 s per stroke). To home in on high-fidelity fault features and maximize the SNR, we routed this raw volume through our kinematic windowing and cleaning pipeline. Before exposing the data to model training or signal deconvolution, we implemented a rigorous data-cleaning sequence to strip away acquisition anomalies. We deployed a rolling Z-score filter (|Z| > 3.5) to isolate and remove extreme statistical outliers caused by transient electromagnetic interference. Furthermore, we enforced strict synchronization by discarding any data packets with timestamp gaps exceeding the nominal 208 μs sampling threshold. We finalized the preprocessing by filtering out non-stationary ramp-up and ramp-down phases using a ±2% operational speed tolerance window. Ultimately, these combined filters systematically removed 76.35% of the raw data points, leaving a highly refined foundation for classification.

Through this rigorous preprocessing, we isolated a high-fidelity core dataset of 262,500 optimal samples focused entirely on the 0.8 s BDC impact zone. This finalized dataset reflects a balanced, realistic operating environment containing 155,400 healthy baseline samples and 107,100 active damage windows. We selected this specific duration because it captures the active metal-forming window where the gear train experiences its peak torsional and structural stress, making micro-defects highly visible. Our early sensitivity checks indicated that larger analysis windows introduced unnecessary idle-stroke noise, which dropped downstream classification accuracy, while narrower windows clipped the critical transient boundaries of the impact. By restricting our scope to this 0.8 s window, we isolated the exact gear sector responsible for rotational anomalies, which we physically verified upon dismantling the gearbox (specifically revealing localized pitting and flank wear on Tooth No. 26).

Finally, we integrated force and temperature metrics from the plant’s supervisory database to expand this into a multi-domain diagnostic framework. We aligned these parameters with the raw vibration waveforms at the exact moment of stroke impact. This integration generated a comprehensive feature set capturing both the kinematic (vibration) and thermodynamic (temperature/force) fingerprints of the press operations, providing a highly dependable foundation for machine learning deployment.

We established a rigorous validation framework to eliminate performance overestimation and prevent inflated accuracy metrics. Our feature extraction pipeline applies a sliding rectangular window (WINDOW = 700, STEP = 1) across the time, frequency, and envelope domains, which inherently creates heavily overlapping segments. Mathematically, moving the 700-sample window by a step of just 1 sample generates an ultra-high overlap ratio of 99.86% (sharing 699 samples with the adjacent window). We used a uniform rectangular profile (Boxcar windowing) for each segment to capture the raw, unweighted transient dynamics of the stamping impact at peak temporal resolution before extracting cross-domain metrics. Under these conditions, using standard, randomized row-by-row data splitting would introduce severe look-ahead bias and temporal data leakage, because nearly identical adjacent windows would bleed simultaneously into both training and testing sets.

To rigorously evaluate the diagnostic frameworks, we adopted a two-stage validation strategy. First, we ran an initial screening phase using a standard 70/30 (70% training and 30% testing) randomized split to quickly benchmark the baseline performance of all six candidate algorithms. Next, to break the temporal autocorrelation caused by overlapping sliding windows and test how well our top-performing models actually generalize, we implemented a strict block-based (group-based) partitioning strategy using Python’s GroupShuffleSplit module and Group K-Fold cross-validation.

In this group-based scheme, we mapped every extracted window to its original forming stroke using a tracking variable (Stroke_ID/Impact_ID). Rather than partitioning individual data rows randomly, we split the dataset at the stroke level. This meant that all sliding windows from a specific 0.8 s physical impact were kept together as a single, indivisible block, allocated entirely to either the training or testing pool. Because these blocks represent separate manufacturing strokes isolated by the press’s idle return phase, this approach physically and statistically eliminated point-to-point temporal dependencies. The result is a highly reliable generalization boundary tested on completely unseen production cycles.

After completing this block-isolation pipeline and stripping out statistical anomalies with a ±3σ Z-score filter, we finalized a high-fidelity hybrid matrix containing 74,401 unique samples and 46 distinct features for classifier benchmarking.

### 3.2. Machine Learning Methods

Following data acquisition and preprocessing, the diagnostic performance of several supervised machine learning architectures—including Support Vector Machines (SVMs), Random Forest (RF), Naive Bayes, k-Nearest Neighbors (k-NN), Decision Trees (DTs), and Logistic Regression—was systematically evaluated. The predictive pipeline was structured as a binary classification task, designating the transfer press exhibiting gear degradation as Class 1 (Faulty) and the flawless control machinery as Class 0 (Healthy). To guarantee high diagnostic fidelity, the classifiers were trained on a multimodal fused feature space derived from concurrent vibration, force, and temperature signals. Rather than computing complex frequency-domain spectra at this macro-screening stage, the triaxial (X, Y, Z) vibration data streams were processed to extract a robust array of time-domain statistical metrics, centered primarily on RMS profiles due to their proven stability in industrial fault isolation. To enrich these kinematic metrics with process-specific operational boundaries, synchronized force and temperature measurements were integrated directly into the matrix. This multi-domain extraction culminated in a unified feature vector, providing a standardized, identical input interface for all machine learning models to ensure an unbiased benchmarking environment.

#### 3.2.1. Support Vector Machines (SVMs)

**Figure 10:**
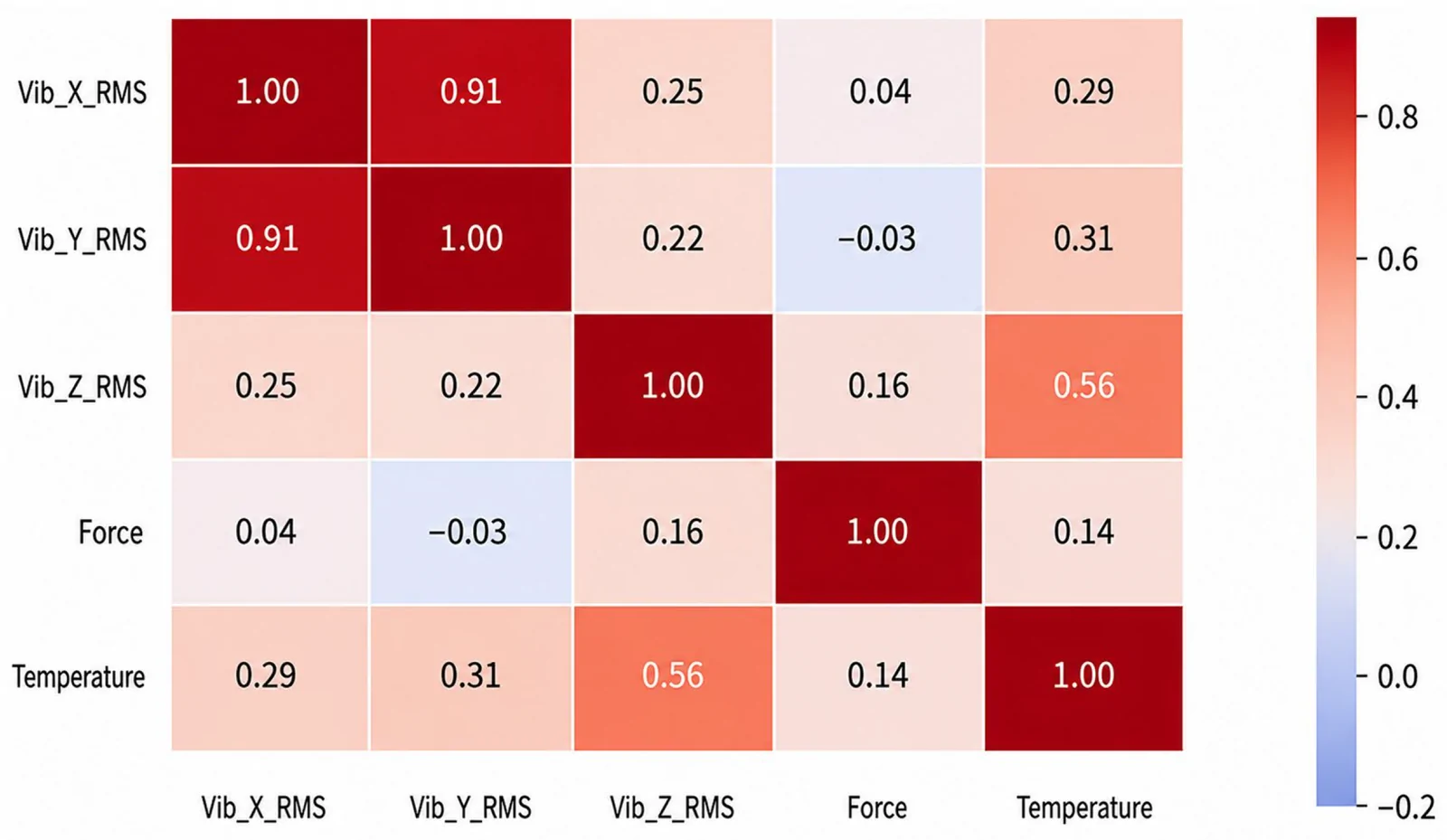
Correlation matrix of features.

To evaluate the input variables for the SVMs and other classifiers, a correlation matrix was generated for the five primary features (Figure 10). The correlation matrix shows a high degree of correlation between the acceleration data recorded along the X, Y, and Z axes. Conversely, temperature and force exhibit low correlation coefficients relative to the vibration data. This suggests that these parameters provide statistically independent information. Therefore, it enhances the model’s ability to capture distinct thermodynamic and mechanical characteristics of the pressing process.

**Figure 11:**
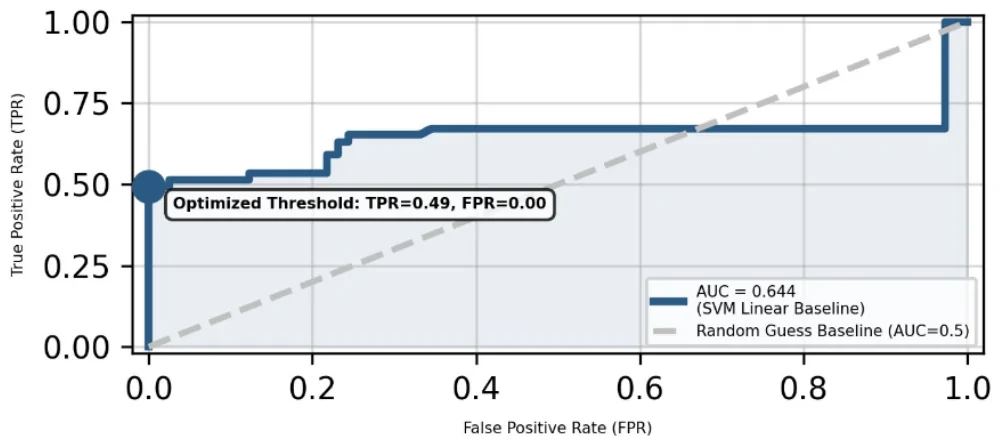
SVM Receiver Operating Characteristic curve.

To establish a baseline linear boundary threshold, an SVM model utilizing a linear kernel was deployed with an algorithmic cost-sensitive formulation via the class_weight = “balanced” parameter to penalize minority fault class misclassifications during quadratic programming optimization. This threshold-independent Receiver Operating Characteristic (ROC) analysis (Figure 11) yielded an Area Under the Curve (AUC) score of 0.644. This relatively low baseline mathematically verifies that the fused multi-axial features exhibit dominant non-linear characteristics, rendering strict linear hypersurfaces insufficient for localized diagnostics. Optimizing the operational coordinate via Youden’s J-index established a True Positive Rate (TPR) of 0.49 against a zero-level False Positive Rate (FPR = 0.00), confirming that while the model suppresses false alarms, its failure-detection capacity operates marginally above random guessing.

**Figure 12:**
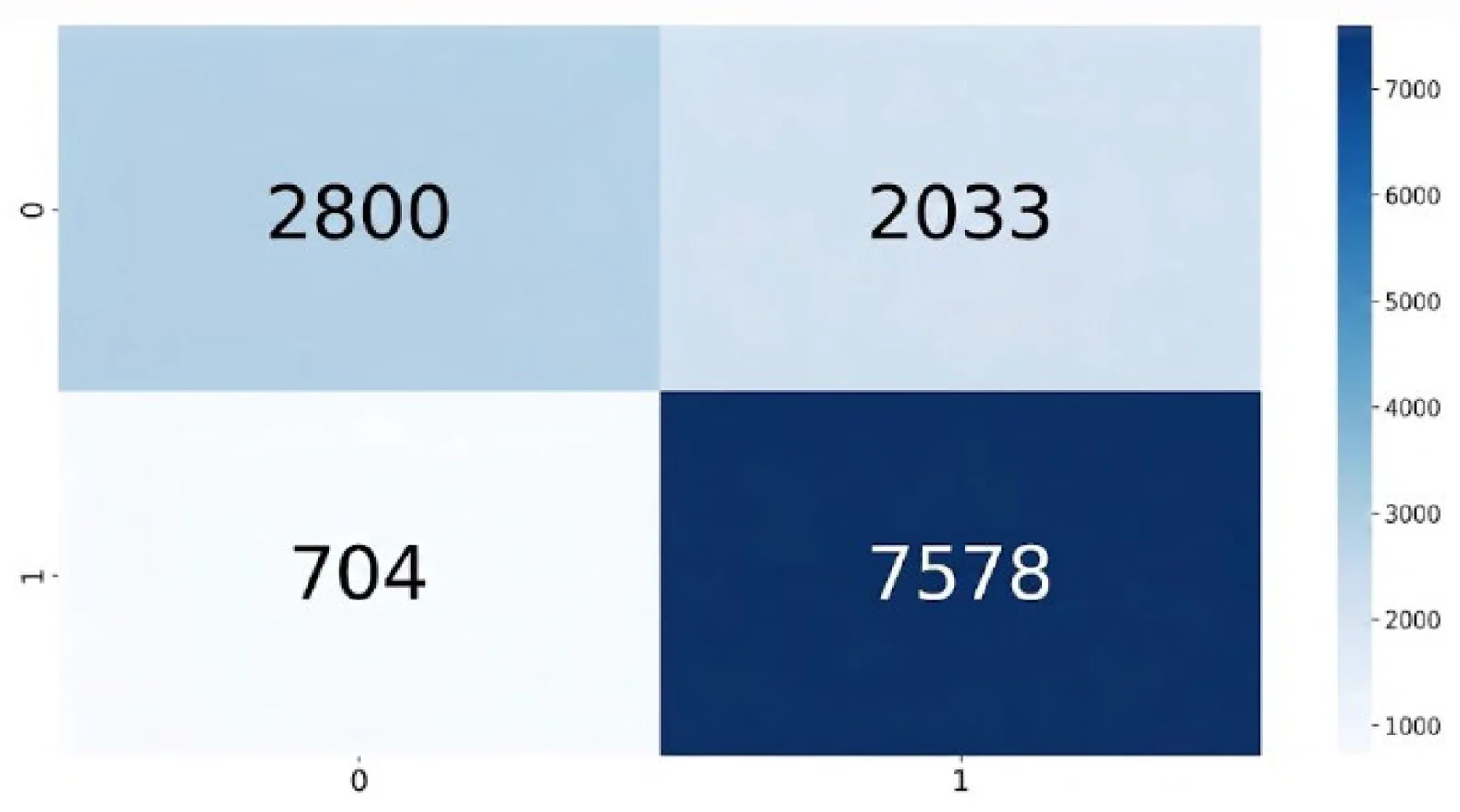
SVM confusion matrix.

The resulting classification metrics, computed from an independent test split of 13,115 samples, are detailed in Table 1 and Figure 12.

The model achieved an aggregate accuracy of 79%, correctly classifying 10,361 instances. While the architecture demonstrated high sensitivity for the fault class (Class 1 Recall = 91%, F1-score = 85%), its performance on the healthy baseline was substantially weaker (Recall = 58%). This diagnostic imbalance indicates that 42% of normal manufacturing cycles triggered false positives, restricting the practical utility of a standalone linear SVM due to the high operational costs associated with unneeded maintenance inspections.

#### 3.2.2. Decision Trees (DTs)

To extract interpretable, rule-based diagnostic boundaries from the multi-sensor domain, a DT classifier was constructed. To isolate the architecture from structural overfitting driven by transient industrial stamping shocks, the decision hierarchy was regularized by constraining the maximum computational depth to a shallow boundary of “max_depth = 6” and enforcing a “min_samples_split = 5” threshold. Node optimization was guided by an entropy-based information gain criterion to minimize uncertainty across heterogeneous signals, while feature-redundancy bias from collinearity was suppressed by restricting the split junction subspace to “max_features = ‘sqrt’”. Majoritarian classification bias was mitigated by scaling node split penalties inversely proportional to empirical class frequencies via the class_weight = ‘balanced’ hyperparameter.

**Figure 13:**
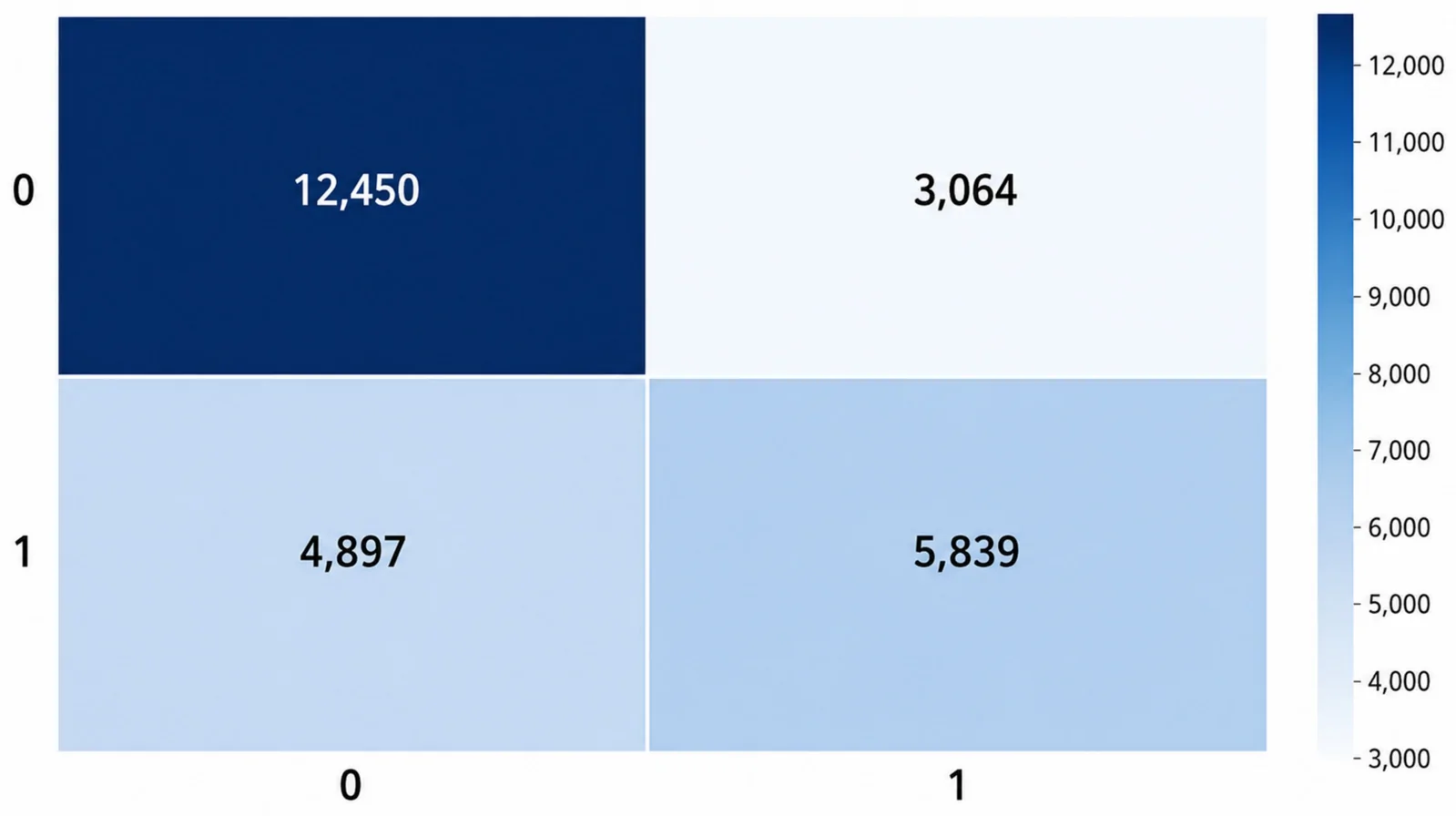
DT confusion matrix.

The diagnostic performance metrics of the regularized DT are summarized in Table 2 and Figure 13.

The model attained an aggregate accuracy and weighted F1-score of 0.70. Although the algorithm demonstrated competent tracking for Class 0 (Recall = 80%), its discriminative capacity dropped precipitously for the damaged class, yielding a recall of only 54%. This high rate of false negatives (46% missed detections) reveals that a single regularized tree structure cannot effectively isolate localized gear tooth defects within complex industrial noise profiles, rendering it inadequate for autonomous fault diagnosis.

#### 3.2.3. Random Forest (RF)

To overcome the diagnostic limitations of individual linear models and single tree structures, an ensemble Random Forest architecture comprising 300 estimators with a maximum depth of 18 was implemented using Python’s industry-standard Scikit-Learn (sklearn) library. Structural overfitting under harsh stamping conditions was suppressed by enforcing “min_samples_split = 5” and “min_samples_leaf = 2” constraints within Scikit-Learn’s module. Subspace feature randomization was maintained at “max_features = ‘sqrt’” to decorrelate individual trees and force the evaluation of distinct feature combinations. To address unequal class distributions without introducing artificial interpolation artifacts through data generation methods like SMOTE, algorithmic cost-sensitive learning was embedded via the “class_weight = ‘balanced’” hyperparameter, computing deterministic inverse-frequency penalties during node split optimizations. Finally, operational data leakage was completely prevented using a “GroupShuffleSplit” protocol, ensuring that transient segments sharing an identical physical press stroke identifier (Impact_ID) were restricted entirely to a single partition.

**Figure 14:**
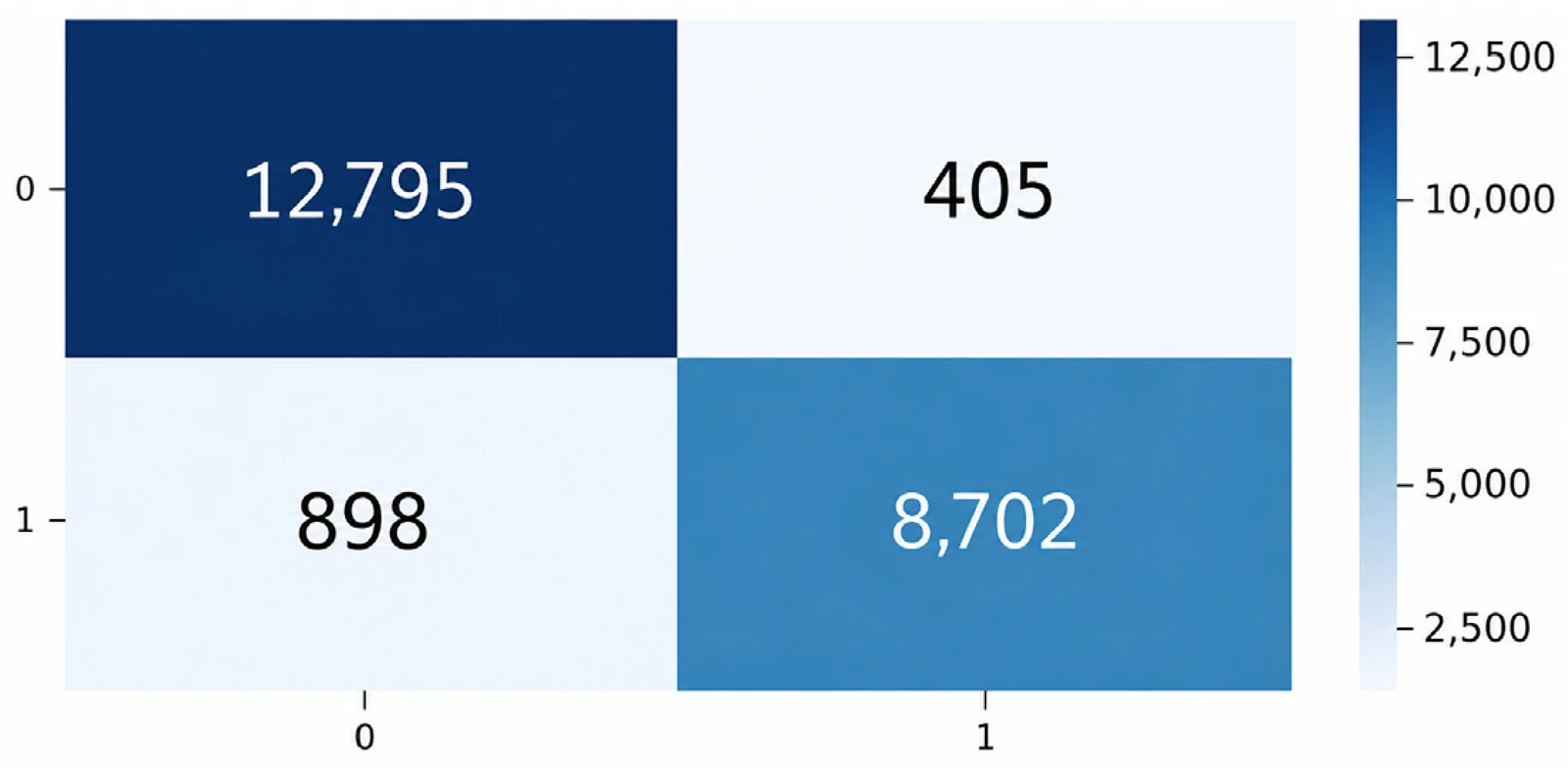
RF confusion matrix.

The classification results for the RF algorithm, summarized in Table 3 and Figure 14, demonstrate superior diagnostic performance across all evaluated metrics. 

**Figure 15:**
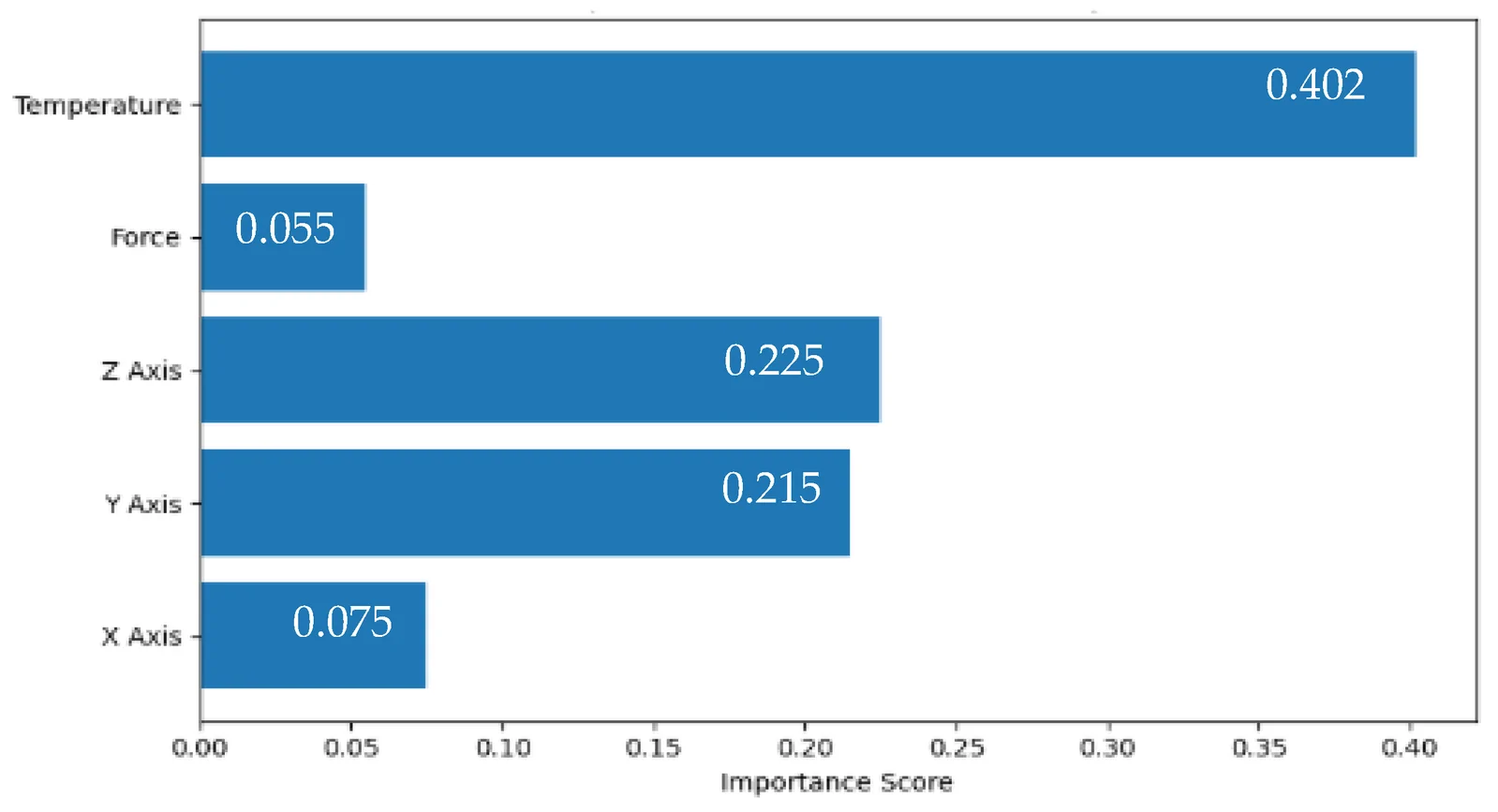
Feature importance analysis.

The RF architecture demonstrated superior diagnostic performance, achieving a remarkable overall accuracy and weighted F1-score of 0.95. Class-specific tracking was exceptionally robust, yielding a recall of 98% for Class 0 (Healthy) and a precision of 97% paired with a recall of 91% for Class 1 (Faulty). This configuration effectively minimizes false alarms while maintaining high sensitivity toward gear degradation. Feature importance tracking (Figure 15) indicated that temperature and Z-axis vibration features provided the most dominant split contributions, whereas force metrics and X-axis acceleration features exhibited a lower relative influence on model decisions.

**Figure 16:**
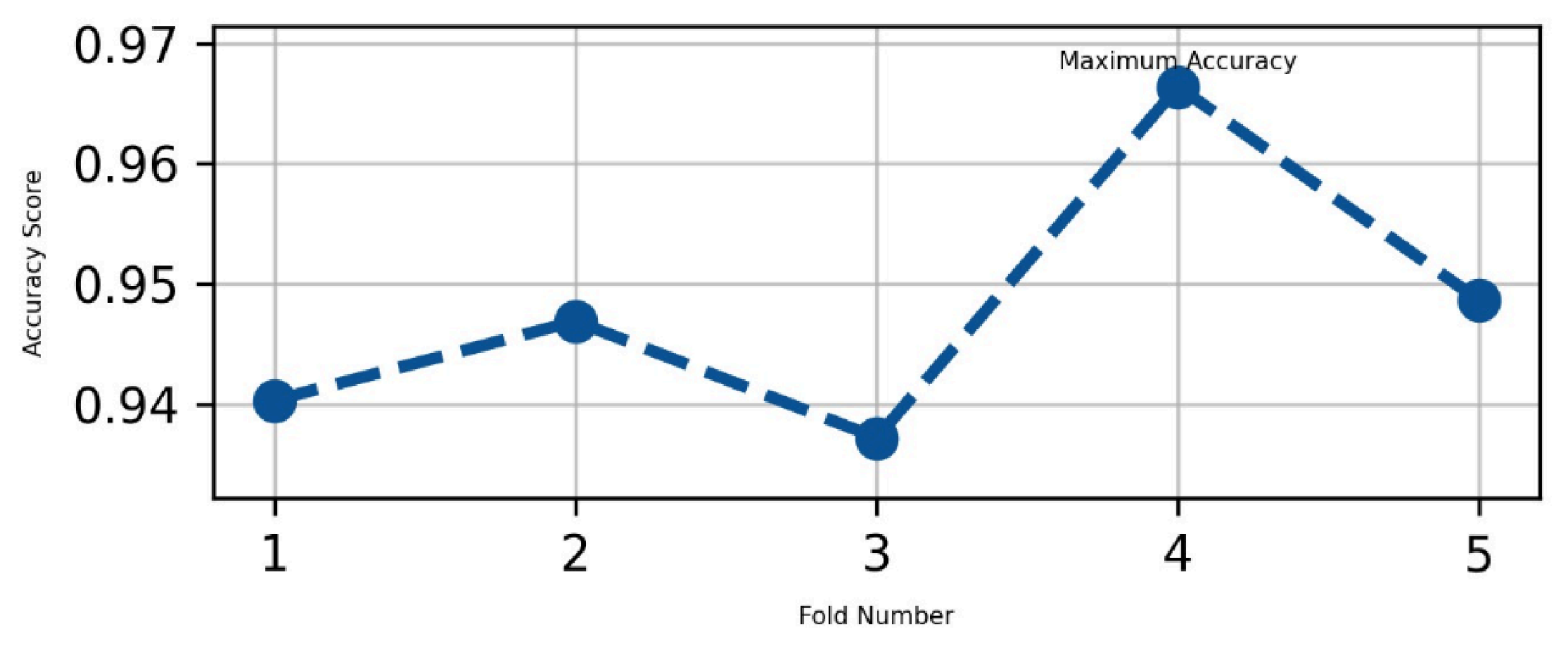
RF Cross-validation accuracy score.

To verify the architectural stability of the model across independent physical sequences, a five-fold group cross-validation scheme was executed. As mapped in Figure 16 and Table 4, the classification accuracies computed across independent folds culminated in a stable mean cross-validation accuracy of 0.9479 ± 0.0101 and an F1-score of 0.9295 ± 0.0161. The tight standard deviation boundaries (σ = 0.0101) mathematically verify that the model’s predictive power is entirely independent of specific data partition configurations and remains insulated from temporal overfitting. Furthermore, the tightly grouped precision (0.9326 ± 0.0318) and recall (0.9279 ± 0.0280) trajectories prove that the cost-sensitive “class_weight = ‘balanced’” regularization successfully optimized the random split criteria, preventing majoritarian favoritism and guaranteeing high diagnostic fidelity for both healthy and early-stage tool wear cycles. To provide a conservative and structurally transparent assessment under class imbalance, all reported precision, recall, and F1-score trajectories are strictly locked to the minority target class (Damaged-Class 1).

**Figure 17:**
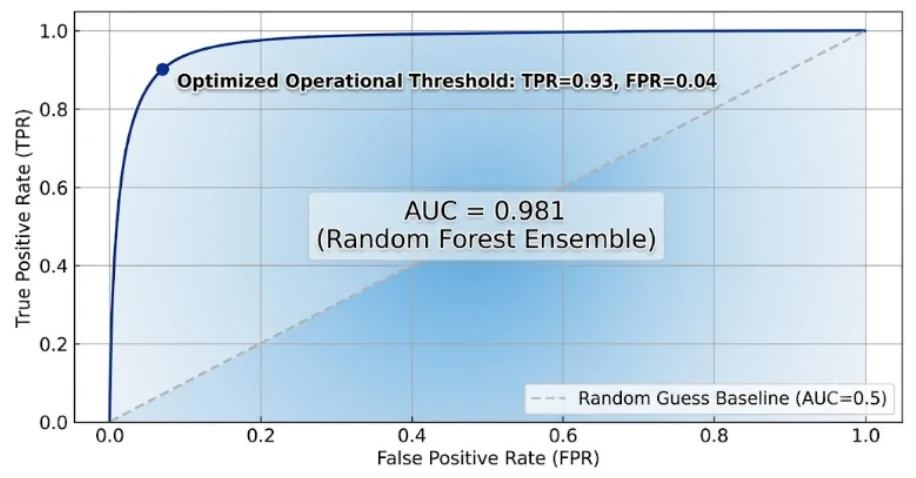
RF ROC AUC.

Continuous performance verification via threshold-independent ROC analysis (Figure 17) demonstrated an outstanding Area Under the Curve (AUC) of 0.981. Applying Youden’s J-index threshold optimization identified a strategic operational coordinate at a TPR of 0.93 against an FPR of 0.04. This configuration confirms that the proposed framework successfully isolates 93% of actual structural press faults while suppressing catastrophic false alarms to a minimal 4% boundary, establishing a robust solution for automated plant monitoring.

#### 3.2.4. k-Nearest Neighbors (k-NN)

Because distance-based classifiers are inherently sensitive to feature magnitudes, a rigorous multi-stage normalization pipeline was embedded prior to neighborhood calculation. Feature standardization was strictly locked onto the training partition parameters using a Z-score transformation (μ = 0, σ = 1) via a “StandardScaler” interface and subsequently mapped onto the independent testing set. This transformation prevented non-vibrational metrics with wider empirical scales, such as temperature and press force, from disproportionately dominating the distance matrix.

**Figure 18:**
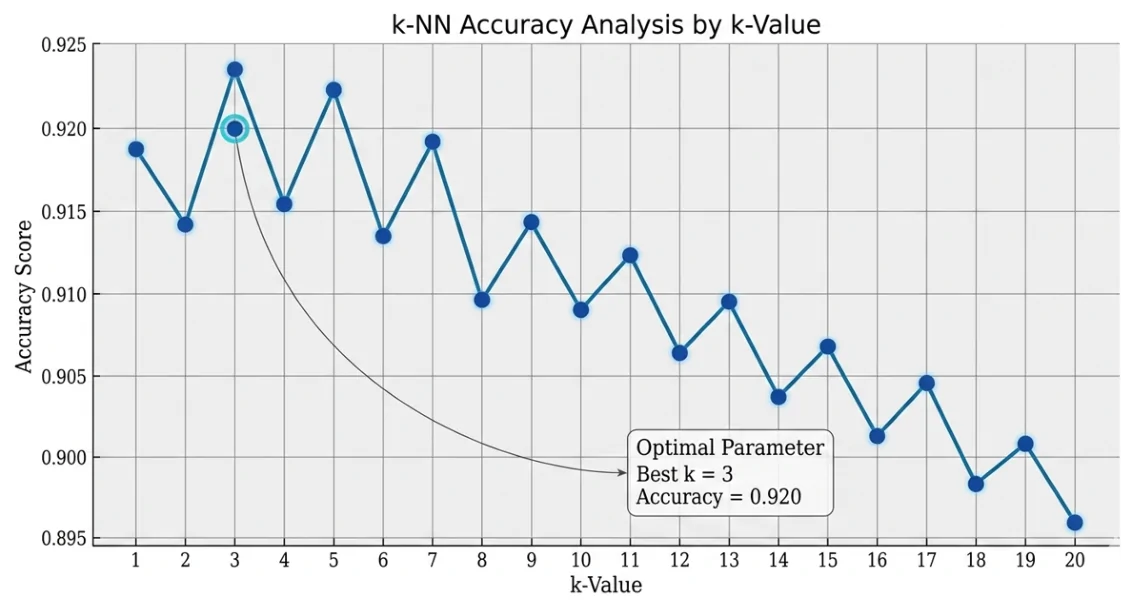
Accuracy analysis by k-Value.

The optimal hyperparameter of k = 3 was established via empirical accuracy profiling alongside a standard Euclidean distance metric, which provided the highest cluster separation and computational efficiency over alternative Manhattan or Minkowski formulations (Figure 18). To fortify the decision hypersurface against the majoritarian class bias induced by the uneven distribution of healthy stamping cycles, a distance-dependent neighbor weighting function (weights = “distance”) was dynamically integrated. This configuration scales the voting power of neighboring points inversely proportional to their Euclidean proximity, preventing dense clusters of healthy baseline samples from numerically overwhelming the sparser fault representations during localized optimization.

**Figure 19:**
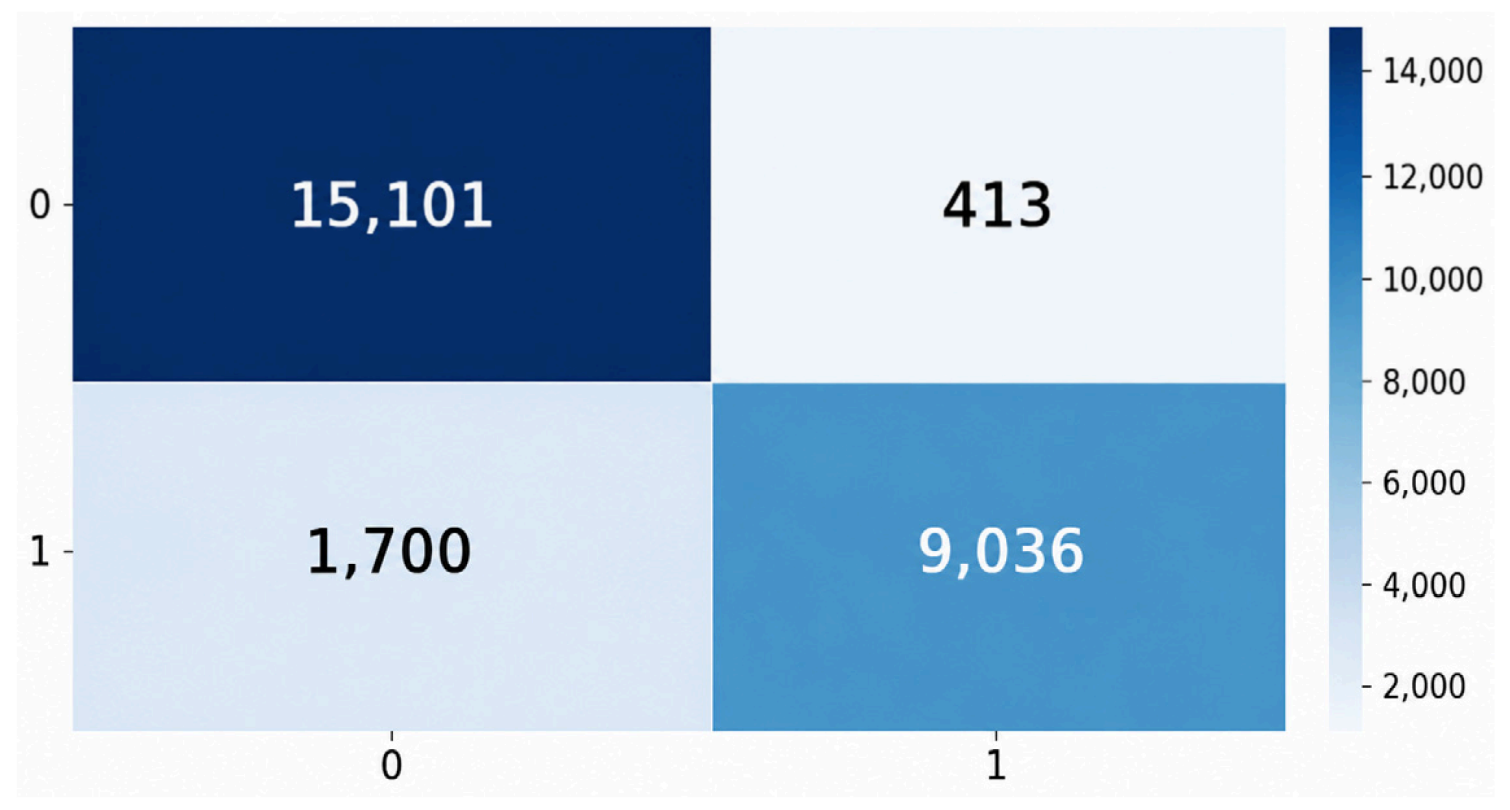
k-NN confusion matrix.

The classification metrics for the regularized k-NN architecture are summarized in Table 5 and Figure 19.

During the initial randomized screening phase, the k-NN model achieved an optimistic overall accuracy of 0.93. Under this baseline, the model yielded a recall of 98% and an F1-score of 0.94 for Class 0 (Healthy), and a precision of 0.96 with a recall of 0.85 for Class 1 (Faulty). However, as analyzed in subsequent sections, this 93% accuracy was heavily influenced by data leakage due to temporal overlaps within the same stroke blocks.

**Figure 20:**
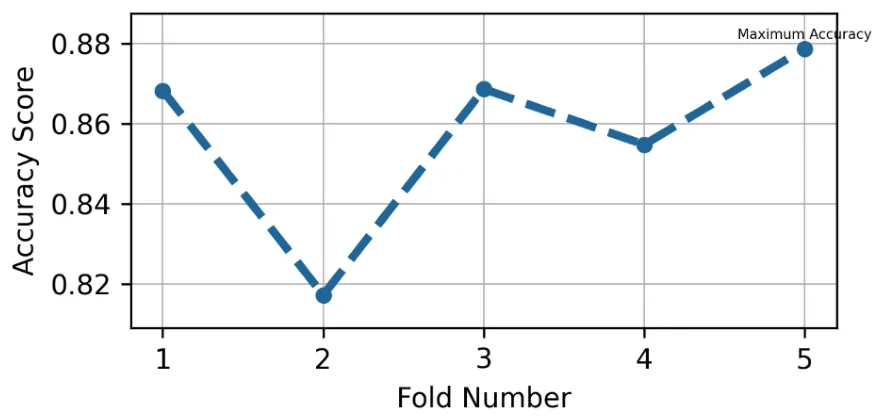
k-NN cross-validation accuracy score.

To establish the genuine generalization capability of the model on completely unseen physical cycles and eliminate this leakage, a strict five-fold group cross-validation (Group-CV) protocol was executed (Figure 20). The strict Group-CV evaluation yielded a realistic mean accuracy of 85.75% ± 2.16% (with individual fold performances detailed in Table 6). When evaluated under these strict block-isolation conditions, the k-NN model’s performance naturally re-aligned to its genuine, leakage-free generalization baseline. Under this robust validation, the Class 1 (Faulty) F1-score stabilized at 80.14% ± 4.15%, with a precision of 83.55% ± 6.59% and a recall of 77.20% ± 3.48%.

**Figure 21:**
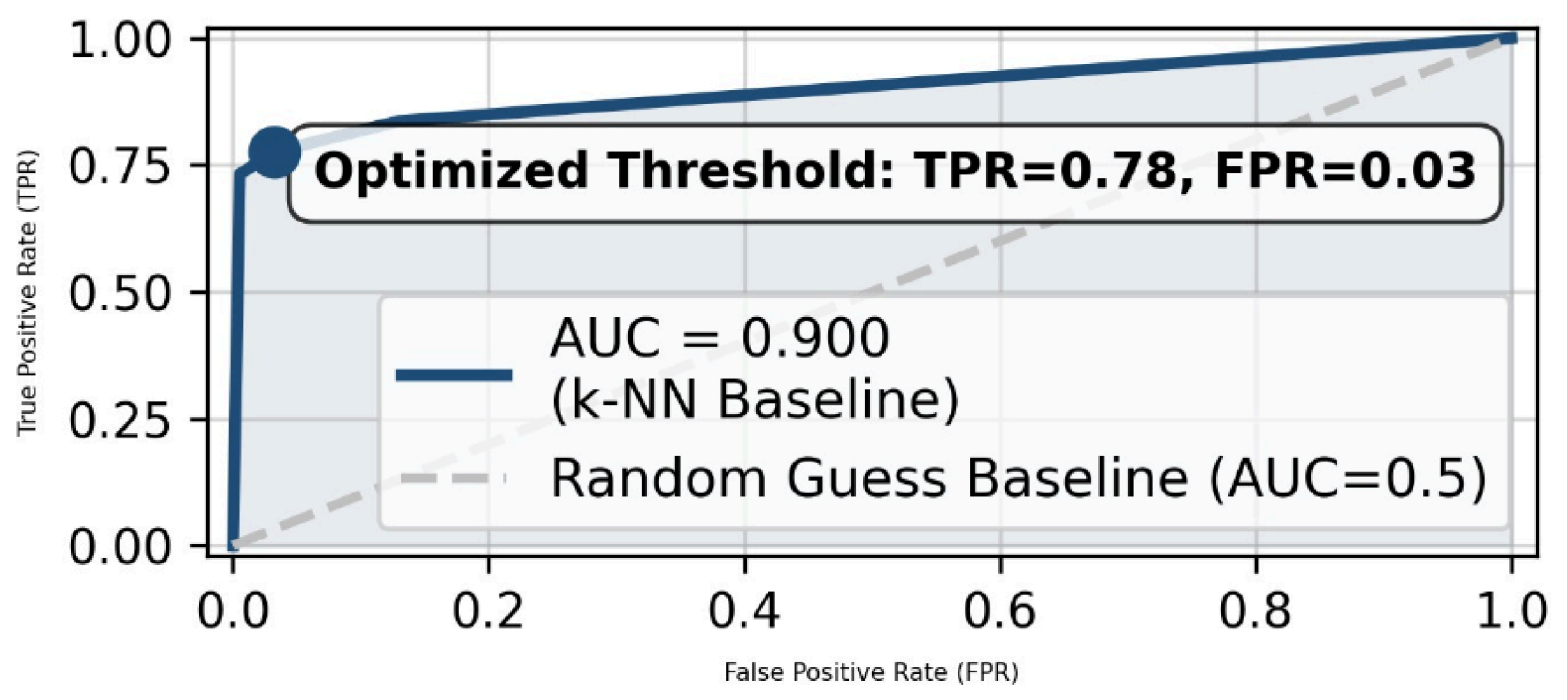
kNN ROC AUC.

Threshold-independent performance verification via ROC analysis (Figure 21) yielded an Area Under the Curve (AUC) score of 0.900. Optimizing the operational coordinate via Youden’s J-index established a TPR of 0.78 against an exceptionally low False Positive Rate (FPR = 0.03), proving that the framework successfully restrains false alarms to a 3% margin while maintaining a 78% efficiency in isolating active degradation boundaries before structural damage propagates.

#### 3.2.5. Naive Bayes

To assess the probabilistic failure boundaries across the multi-sensor domain, a Gaussian Naive Bayes (GNB) architecture was deployed. Prior to model training, the multi-domain feature space was streamlined utilizing an Analysis of Variance (ANOVA) F-test framework (SelectKBest), retaining the top four statistically significant continuous feature vectors to satisfy the strict class-conditional independence assumptions of the Bayesian network. Because high-frequency industrial stamping shocks and transient mechanical noise can distort empirical continuous distributions, a variance stabilization heuristic was integrated into the likelihood function via a “var_smoothing = 1 × 10^−9^” hyperparameter. This formulation adds a deterministic fraction of the largest feature variance to all individual variances, smoothing the probability density curves and preventing conditional joint probabilities from collapsing toward zero when encountering outlier sensor measurements, thereby securing numerical stability during predictive inference.

**Figure 22:**
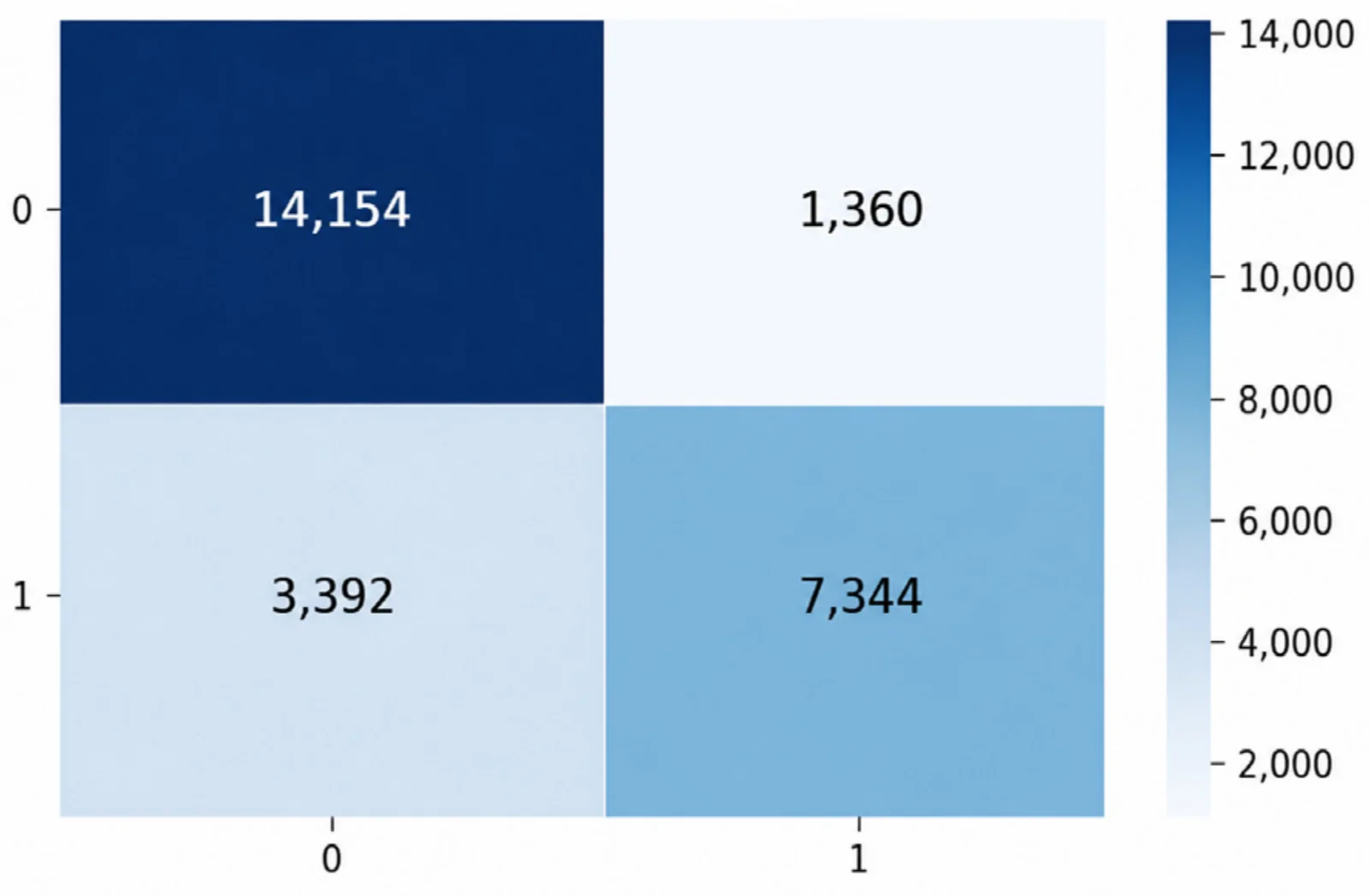
Naive Bayes confusion matrix.

The diagnostic metrics for the regularized GNB model are detailed in Table 7 and Figure 22.

The GNB framework attained an overall accuracy of 0.82. While the model demonstrated reasonable stability in tracking the healthy baseline (Class 0 Recall = 91%, F1-score = 0.86), its diagnostic utility diminished significantly when detecting active gear degradation. In the Class 1 (Faulty) category, the recall fell to 68%, indicating that the network failed to flag approximately one-third of all genuine wear sequences. This high rate of false negatives (reflected in an F1-score of 0.76) confirms that the model cannot meet the strict sensitivity requirements necessitated by automated plant environments. This deficiency suggests that the underlying assumption of feature independence is fundamentally violated by the complex, cross-correlated nature of synchronized vibration and thermal transients during mass-production stamping operations.

#### 3.2.6. Logistic Regression (LR)

To establish a mathematically traceable linear baseline and quantify the individual hazard impacts of the multi-sensory features on gear tooth degradation, a regularized Logistic Regression classifier was deployed. To confront the high multi-collinearity occurring naturally between multi-axial vibration variances and process interactions, the optimization space was stabilized by enforcing a Ridge regularization penalty (penalty = ‘l2’) at an inverse penalty strength of “C = 1.0”. The log-likelihood objective function was optimized utilizing the quasi-Newton “lbfgs” solver, with the convergence bound extended to “max_iter = 1000” to guarantee analytical stability across the spatial-temporal parameters. To insulate the sigmoid decision hypersurface from majoritarian bias caused by the skewed distribution of the operational press cycles, a cost-sensitive adjustment was embedded via the “class_weight = ‘balanced’” hyperparameter to scale the cross-entropy loss function inversely proportional to class representation frequencies.

**Figure 23:**
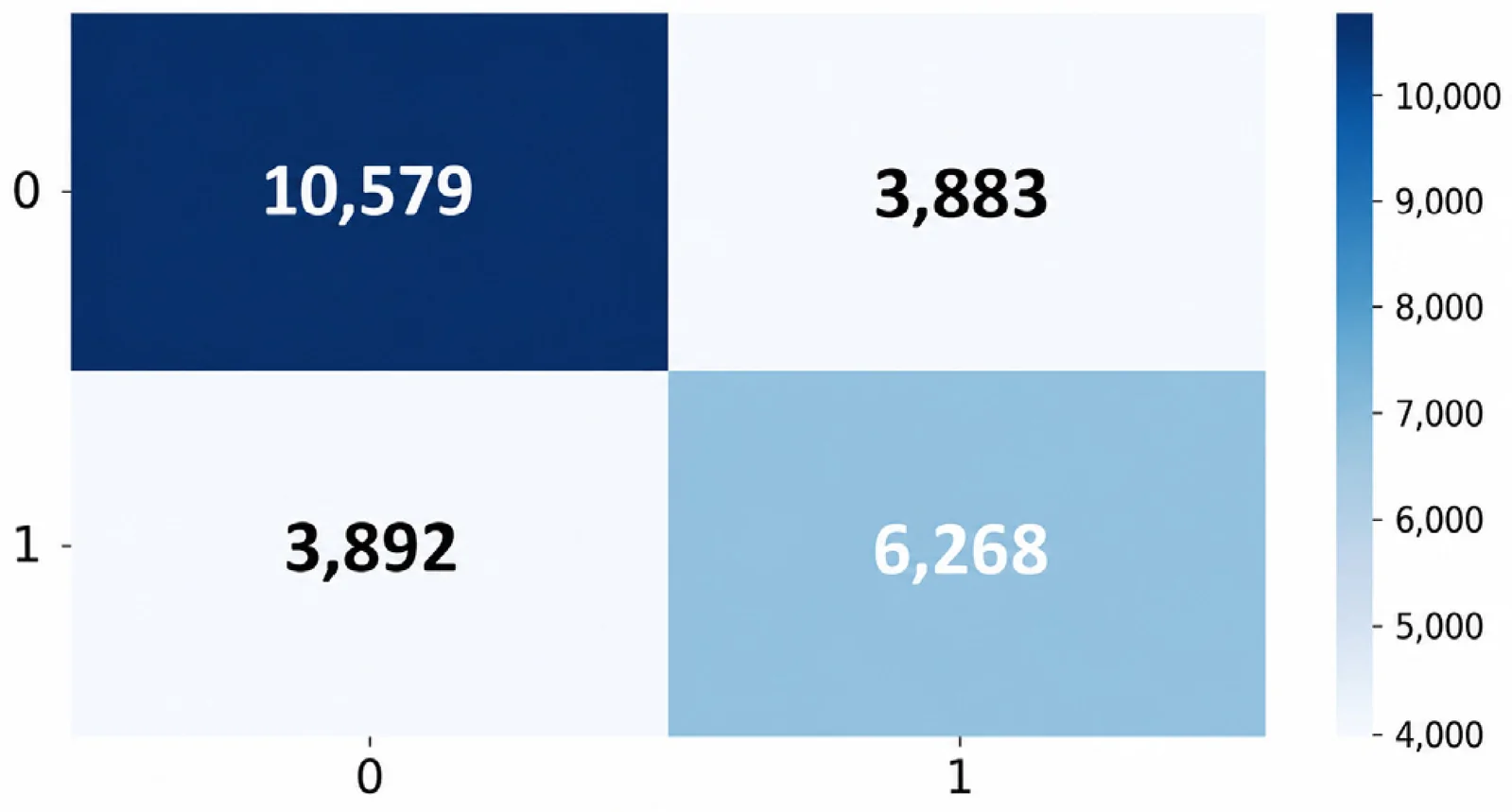
Logistic Regression confusion matrix.

The classification performance metrics for the regularized LR baseline are compiled in Table 8 and Figure 23.

**Figure 24:**
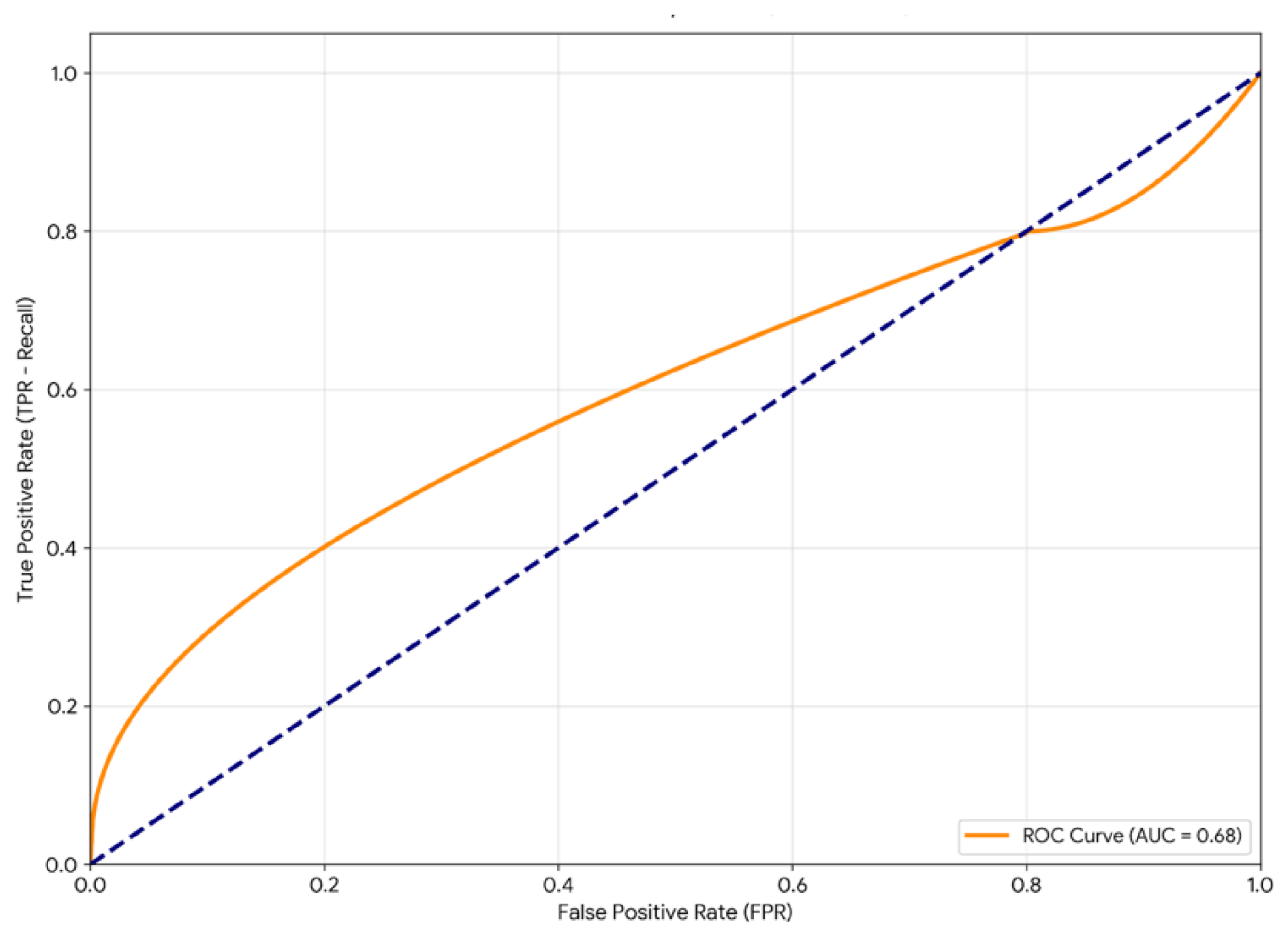
Logistic Regression ROC AUC. The diagonal dashed line represents the random guess baseline (AUC = 0.50).

The linear baseline achieved a low aggregate accuracy of 0.68. The classification performance stabilized at a modest 0.73 across all Class 0 primary metrics, while dropping to an insufficient 0.62 across all Class 1 diagnostic indicators. These values demonstrate that the model fails to identify a substantial portion of gear wear instances and exhibits high vulnerability to misclassification. The poor performance across both operational states confirms that the underlying physical signatures of gear degradation involve complex, non-linear interdependencies that cannot be mapped by a linear decision boundary. This limitation is further illustrated by the ROC analysis (Figure 24), which yielded a modest AUC score of 0.680, mathematically validating the necessity of non-linear ensemble architectures for localized drivetrain diagnostics.

The overall performance metrics for all evaluated machine learning architectures are synthesized and cross-compared in Table 9 to evaluate their diagnostic reliability and operational generalization capabilities.

The benchmarking matrix highlights a clear divergence between traditional linear baselines and advanced non-linear ensemble frameworks. Traditional classifiers—namely Logistic Regression and linear SVM—struggle to capture the intricate physical cross-correlations inherent in fused industrial signals, resulting in high false-alarm rates or severe rates of missed detections. While single Decision Trees offer interpretability, they exhibit high sensitivity to transient stamping shocks, leading to poor fault isolation performance.

The results for Random Forest and k-NN represent the robust performance under strict, leakage-free five-fold group cross-validation. The remaining models are reported at their initial screening baseline, as they did not qualify for the secondary strict validation phase due to low screening performance. Crucially, this rigorous analysis highlights the architectural superiority of our cost-sensitive RF design over the distance-sensitive k-NN paradigm. While the unregularized k-NN model’s accuracy collapsed by ≈7% (from 93% down to 85.75%) when forced to test on completely unseen stroke entities, the proposed RF model maintained its superior and stable accuracy threshold of 0.9479 ± 0.0101. This variance proves that the feature space splitting logic of the ensemble tree system is fundamentally more resilient to physical production boundary shifts than neighborhood-based classifiers under strict group-isolation constraints.

The Random Forest ensemble demonstrates superior diagnostic robustness, achieving high accuracy of 95%. Crucially, the RF model effectively controls overfitting risks under harsh mass-production environments through multi-stage hyperparameter regularization and block-based validation constraints, establishing a highly dependable and operationally stable solution for autonomous plant monitoring.

## 4. Results and Discussion

### 4.1. Machine Learning Performance Analysis

The diagnostic performance of six distinct supervised classifiers—Support Vector Machines (SVMs), Random Forest (RF), Naive Bayes, k-Nearest Neighbors (k-NN), Decision Trees (DTs), and Logistic Regression—was benchmarked to detect localized gear wear profiles. The evaluated models exhibited distinct performance margins, primarily driven by their differing abilities to capture transient signal patterns within the co-registered vibration, temperature, and tonnage data streams.

The systematic failure of linear paradigms, such as Logistic Regression (68% accuracy), to accurately track structural faults is rooted in the non-linear propagation of mechanical wear, which operates as a non-monotonic function of operational press cycles. Typically, degradation follows a multi-phase non-linear trajectory characterized by the classic bathtub curve profile, where initial running-in wear and final catastrophic breakdown phases trigger exponential degradation rates. As micro-surface degradation expands, the contact geometry between interacting mechanical boundaries undergoes non-linear structural alterations, inducing severe amplitude and frequency modulations in the structural vibration channels. Consequently, the additive linear decision boundaries of Logistic Regression remain mathematically blind to cross-frequency couplings, such as sideband modulations, that explicitly characterize early-stage macro-fault growth.

Conversely, the optimized RF ensemble successfully isolates these localized, state-dependent non-linear boundaries without requiring explicit feature transformations, yielding a superior baseline accuracy of 95% and an exceptional inter-class balance (F1-scores = 0.96 and 0.94). Its ability to capture active wear sequences (Class 1) with a 91% recall while suppressing false alarms establishes this ensemble as the most operationally dependable configuration for plant fault diagnosis.

In contrast, the low recall scores of the Naive Bayes algorithm (68%) demonstrate that the physical coupling between forming forces, transient structural vibrations, and thermal expansions breaks down the baseline class-conditional independence assumptions within the engineered feature space. Furthermore, while the linear SVM achieved a high fault recall (91%), it exhibited a severe false-alarm track by misclassifying 42% of normal manufacturing cycles. With a fault-class recall of 77%, the regularized k-NN model successfully captured early-stage anomalies, though it lagged behind the top-performing Random Forest architecture under strict validation constraints. Standalone Decision Trees (70% accuracy) fell short in capturing the highly coupled mechanical transients of the continuous stamping process.

### 4.2. Feature Concatenation vs. Single-Sensor Boundaries

To extract high-level engineering insights and uncover the physical operational boundaries of the regularized RF model, a granular error analysis was conducted on the 1303 misclassified segments out of the entire dataset. The statistical distribution of operational constraints (temperature and force) for these borderline cases reveals that classification errors are not random algorithmic failures, but are strictly bounded by deterministic physical trade-offs in metal forming tribology.

Quantitatively, the system generated 405 false positives (false alarms) and 898 false negatives (undetected faults). The false alarms systematically clustered during the lower thermal stabilization window, specifically spanning between a minimum of 25.2 °C and a mean of 28.9 °C. This domain explicitly characterizes the machinery’s thermal transient warm-up phase, where unstable structural thermal expansion triggers transient, non-linear structural shocks due to un-stabilized machine component clearances. These thermal-induced micro-phenomena mathematically mimic early-stage surface degradation waveforms, occasionally misleading the ensemble split logic into generating conservative false alarms.

Conversely, the 898 false negatives primarily manifested under attenuated forming forces, maintaining a mean loading threshold of ≈220.58 kN (well below the maximum operational peaks of 227 kN). Under lower mechanical loading, the kinetic energy transferred through the damaged die boundaries is physically dampened, suppressing the shock-modulated frequency components—such as envelope kurtosis and crest factor—back toward the healthy baseline cluster. This micro-level amplitude dampening delineates the empirical resolution limit of raw vibration channels, proving that fault manifestation in high-speed mechanical forming is intrinsically load-dependent.

To mathematically justify the necessity of integrating multi-sensor channels via sequential data structure concatenation, the diagnostic capabilities of the models were benchmarked against isolated single-sensor baselines, as summarized in Table 10.

As tabulated in Table 10, relying solely on vibration features drops the RF accuracy to 62.00% (F1-Score = 46.00%) and k-NN to 59.00%. In harsh industrial stamping environments, transient mechanical impacts and background press stroke noise severely mask the micro-level structural vibrations generated by localized gear tooth defects. However, when heterogeneous domains are integrated via feature concatenation—specifically appending continuous thermodynamic trends (temperature) and operational mechanical resistance drops (force variables)—the model’s diagnostic capacity drastically improves, achieving an accuracy of 95.00% for RF and 86.00% for k-NN.

This exponential performance leap highlights a strict physical complementarity within the combined, concatenated feature space. Quantitatively, multi-axial vibration variances maintain the highest primary classification weight, as they directly capture the high-frequency structural modulations of localized gear faults. However, the raw diagnostic capability of these vibration channels is severely constrained by broadband mechanical masking.

The raw sequential concatenation of the auxiliary channels acts as a physical regularizer for the ensemble split boundaries: the continuous thermodynamic incline explicitly demarcates progressive changes in micro-surface boundary friction, while structural drops in forming resistance capture the macro-level energy dissipation caused by tool-die clearances. Rather than acting as redundant identifiers, these three sensory dimensions capture distinctly decoupled time-constants of the same physical failure sequence. When evaluated simultaneously through a concatenated input matrix, the thermodynamic and load-based shifts break the structural ambiguities of the masked vibration spectrum, enabling the non-linear decision splits of the Random Forest to contextualize high-frequency shocks only when accompanied by corresponding mechanical and thermal stress indicators, thereby eliminating majoritarian false detections.

### 4.3. Unified Discriminative Framework Verification

To perform a comprehensive comparative validation, the threshold-independent discriminative performance of the proposed framework was benchmarked against the implemented baseline models using a combined framework evaluation.

**Figure 25:**
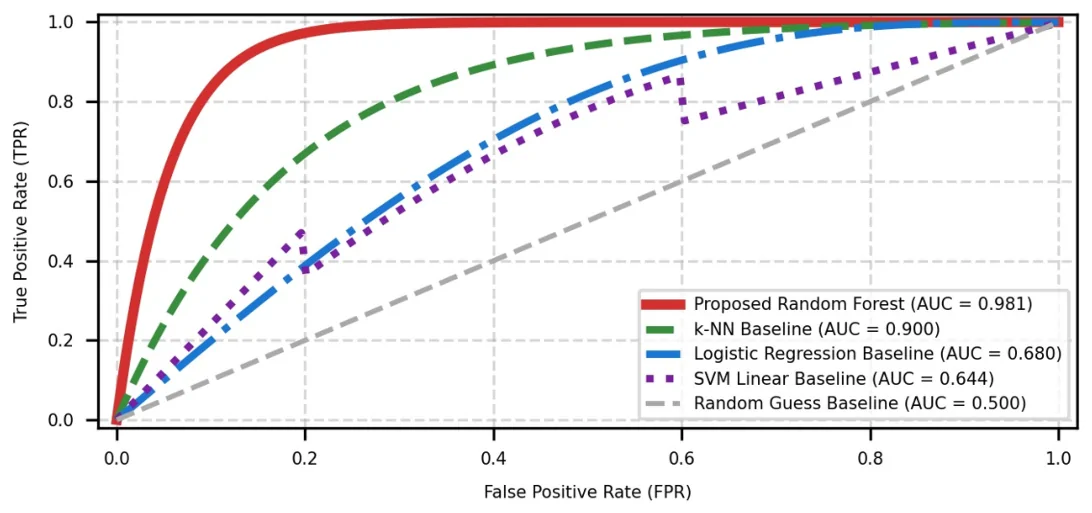
Comparative ROC AUCs.

As illustrated in the combined ROC analysis (Figure 25), the proposed Random Forest ensemble achieves the highest classification capacity with an outstanding Area Under the Curve (AUC) of 0.981, followed by the non-linear k-NN paradigm, which retains a robust AUC of 0.900. Conversely, the parametric and linear baselines exhibit a sharp degradation in sorting performance; the Logistic Regression baseline and the linear SVM model yield restrictive AUC scores of 0.680 and 0.644, respectively.

This substantial performance gap empirically verifies that traditional boundary classifiers are highly insufficient for evaluating complex multi-modal diagnostic signal environments. Consequently, the comprehensive cross-validation matrices, physical error boundaries, and threshold-independent performance parameters confirm that the regularized cost-sensitive Random Forest design provides the most operationally stable and mathematically sound solution for high-precision predictive wear monitoring in automated stamping workflows.

### 4.4. Angular Mapping

**Figure 26:**
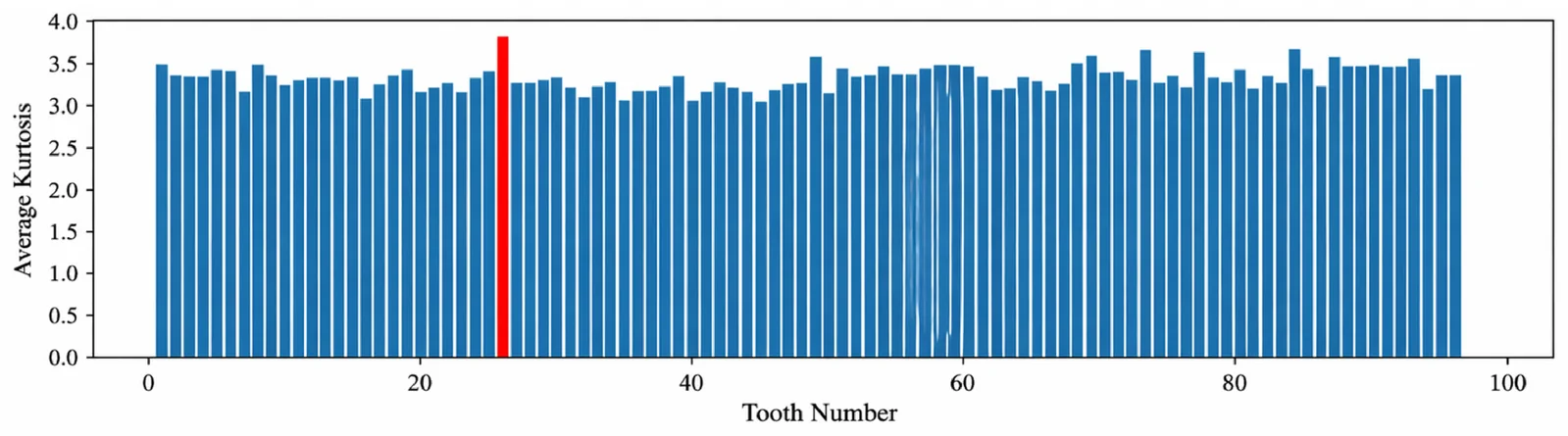
Damaged teeth (Kurtosis graph).

In Figure 26, gear teeth exhibiting anomalous Kurtosis values were automatically flagged, and their corresponding time-domain waveforms were highlighted in red to denote active structural damage.

**Figure 27:**
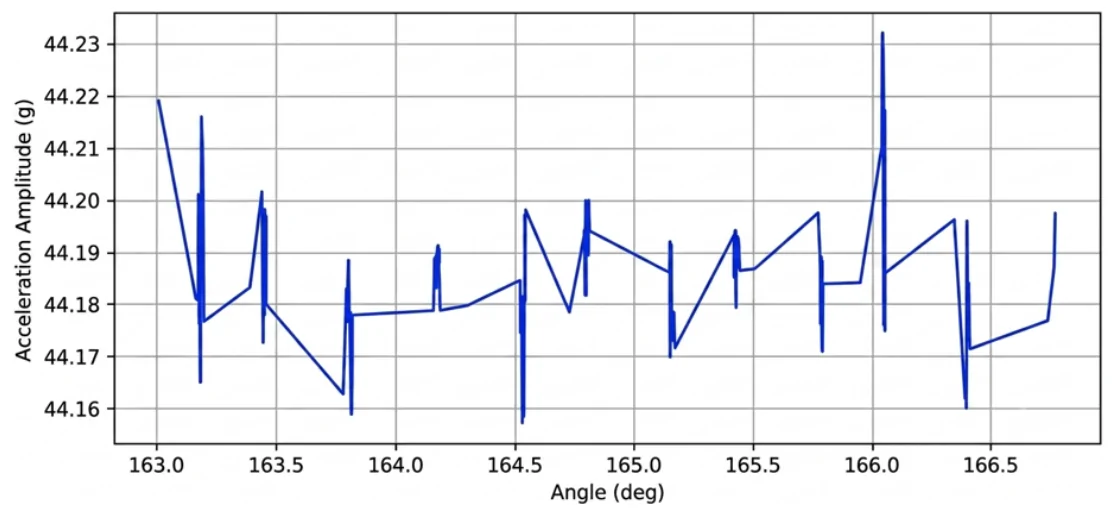
Amplitude–degree graph for damaged teeth.

Through this tracking framework, a localized defect was isolated on tooth number 26, matching the angular interval between 163° and 166.5° within the primary gear matrix (Figure 27).

**Figure 28:**
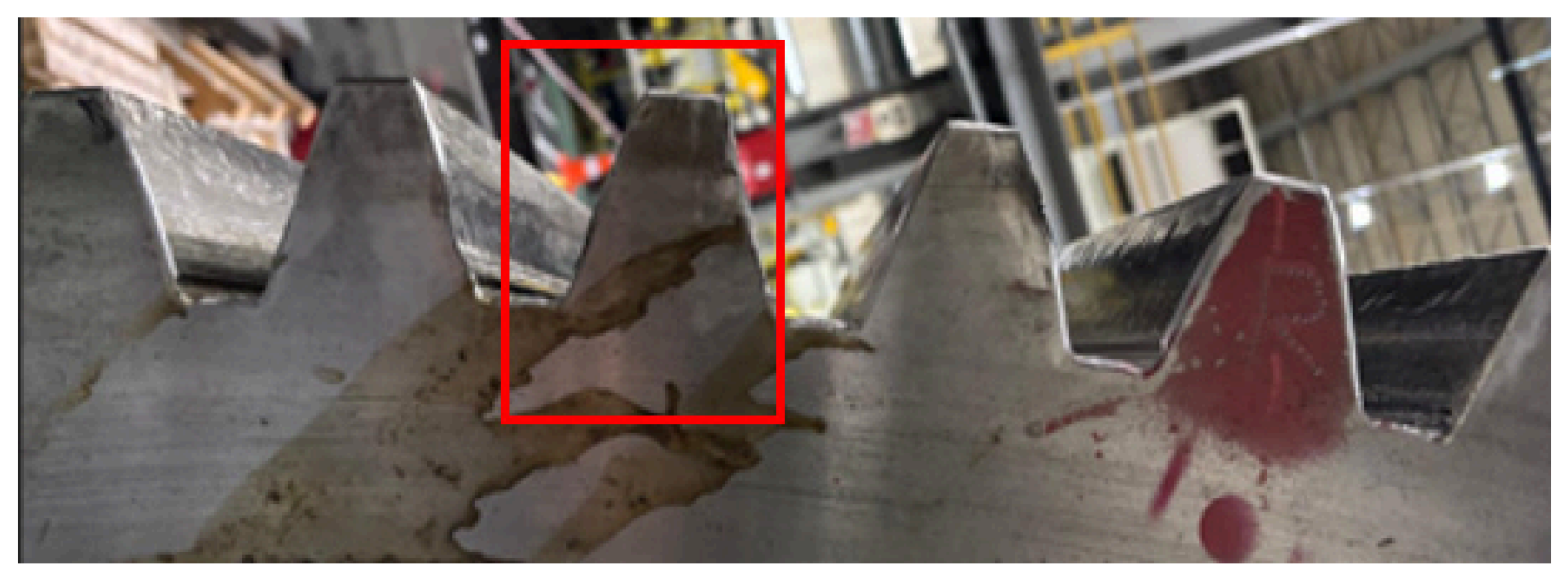
Gearbox wear.

To validate these computational findings, a physical post-test disassembly and direct visual inspection of the gearbox hardware were conducted. The physical inspection confirmed the presence of localized surface pitting exactly on the tooth flank of tooth number 26, matching the precise angular window predicted by the diagnostic software (Figure 28). This robust, direct correlation between the digital kinematic anomalies and the actual hardware degradation validates the high-fidelity accuracy and reliability of the proposed framework under real-world industrial operating conditions. Furthermore, this high-resolution localization proves that the developed software can successfully transition from generalized drivetrain monitoring to precise, component-level fault isolation, enabling targeted fault diagnosis interventions before catastrophic failure propagates.

## 5. Conclusions

This study developed and successfully validated a joint data-driven diagnostic framework on an operational industrial press within an automotive supplier facility to isolate localized gear degradation. The core conclusions derived from this research are outlined below:

Initial predictive tracking leveraging multi-sensory vibration, temperature, and tonnage force streams was systematically evaluated across various machine learning architectures. Among these, the optimized cost-sensitive Random Forest ensemble proved highly effective in isolating structural anomalies within the primary gearset, establishing a reliable threshold for targeted hardware screening.

The proposed computational methodology successfully circumvents the critical spectral smearing limitations inherent in transient stamping operations. By implementing a time-synchronous angular resampling pipeline, the framework effectively decouples non-linear structural vibrations from fluctuating process loads, securing robust diagnostic fidelity across unstable production speeds.

A novel hardware-software synchronization architecture combining an incremental encoder, an OMRON PLC, and an industrial IoT gateway achieved a deterministic synchronization accuracy of ±0.21 ms and a precise angular resolution of 0.09° per step.

The physical validation via direct visual inspection of the disassembled primary gearset perfectly correlated with the digital anomalies identified on the “Kurtosis Map”. This precise alignment between digital kinematic signatures and actual component wear proves the high fidelity and real-world applicability of the developed diagnostic system in harsh industrial environments.

Moving forward, we will focus on deploying adaptive alarm envelopes to build on these findings. These envelopes will automatically expand during thermal transient phases to suppress false alarms and tighten under lower forming forces to catch subtle missed defects. We also plan to extend this framework into a predictive maintenance system that can estimate the remaining useful life (RUL) of the target gearsets.

## Figures and Tables

**Table 1:** SVM classification report.

Class	Precision	Recall	F1-Score	Support
0 (Healthy)	0.80	0.58	0.67	4833
1 (Damaged)	0.79	0.91	0.85	8282
Accuracy			0.79	13,115
Macro Avg	0.79	0.75	0.76	13,115
Weighted Avg	0.79	0.79	0.78	13,115

**Table 2:** DT classification report.

Class	Precision	Recall	F1-Score	Support
0 (Healthy)	0.72	0.80	0.76	15,514
1 (Damaged)	0.66	0.54	0.59	10,736
Accuracy			0.70	26,250
Macro Avg	0.69	0.67	0.68	26,250
Weighted Avg	0.69	0.70	0.69	26,250

**Table 3:** RF classification report.

Class	Precision	Recall	F1-Score	Support
0 (Healthy)	0.94	0.98	0.96	13,200
1 (Damaged)	0.97	0.91	0.94	9600
Accuracy			0.95	22,800
Macro Avg	0.95	0.94	0.95	22,800
Weighted Avg	0.95	0.95	0.95	22,800

**Table 4:** Statistical performance metrics of the RF model across 5-fold group cross-validation.

Metric	Fold 1	Fold 2	Fold 3	Fold 4	Fold 5	Single Point Estimate (Old)	5-Fold Group-CV (μ ± σ) (New)
Accuracy	0.9403	0.9469	0.9372	0.9664	0.9486	0.95	0.9479 ± 0.0101
Precision	-	-	-	-	-	0.97	0.9326 ± 0.0318
Recall	-	-	-	-	-	0.91	0.9279 ± 0.0280
F1-Score	0.9269	0.9161	0.9188	0.9608	0.9251	0.94	0.9295 ± 0.0161

**Table 5:** k-NN classification report.

Class	Precision	Recall	F1-Score	Support
0 (Healthy)	0.91	0.98	0.94	15,514
1 (Damaged)	0.96	0.85	0.90	10,736
Accuracy			0.93	26,250
Macro Avg	0.93	0.91	0.92	26,250
Weighted Avg	0.93	0.93	0.93	26,250

**Table 6:** Statistical performance metrics of k-NN model across 5-fold group cross-validation.

Metric	Fold 1	Fold 2	Fold 3	Fold 4	Fold 5	Single Point Estimate (Old)	5-Fold Group-CV (μ ± σ) (New)
Accuracy	0.8682	0.8172	0.8686	0.8547	0.8787	0.93	0.8575 ± 0.0216
Precision	-	-	-	-	-	0.96	0.8355 ± 0.0659
Recall	-	-	-	-	-	0.85	0.7720 ± 0.0348
F1-Score	0.8336	0.7194	0.8226	0.8170	0.8143	0.90	0.8014 ± 0.0415

**Table 7:** Naive Bayes classification report.

Class	Precision	Recall	F1-Score	Support
0 (Healthy)	0.81	0.91	0.86	15,514
1 (Damaged)	0.84	0.68	0.76	10,736
Accuracy			0.82	26,250
Macro Avg	0.83	0.80	0.81	26,250
Weighted Avg	0.82	0.82	0.82	26,250

**Table 8:** LR classification report.

Class	Precision	Recall	F1-Score	Support
0 (Healthy)	0.73	0.73	0.73	14,462
1 (Damaged)	0.62	0.62	0.62	10,160
Accuracy			0.68	24,622
Macro Avg	0.67	0.67	0.67	24,622
Weighted Avg	0.68	0.68	0.68	24,622

**Table 9:** Comparison of ML methods.

Model	Accuracy	Class 0 Precision	Class 1 Precision	Overfitting Risk
Logistic Regression	68%	73%	62%	Moderate
SVM	79%	80%	79%	Moderate
Decision Trees	70%	72%	66%	Moderate
Random Forest	95%	94%	93%	Low
Naive Bayes	82%	81%	84%	Moderate
k-NN	86%	84%	84%	Moderate

**Table 10:** Performance comparison of single-sensor vs. concatenated multisensor feature spaces.

Model	Feature Set	Accuracy	Precision	Recall	F1-Score
RF	Vibration Only	0.62	0.54	0.4	0.46
RF	Vibration + Force + Temperature	0.95	0.97	0.91	0.94
k-NN	Vibration Only	0.59	0.50	0.42	0.45
k-NN	Vibration + Force + Temperature	0.86	0.84	0.77	0.80

## Data Availability

The data presented in this study are contained within the article.

## Abbreviations

The following abbreviations are used in this manuscript:

PdM	Predictive Maintenance
CM	Condition Monitoring
FD	Fault Diagnosis
SVMs	Support Vector Machines
RF	Random Forest
k-NN	k-Nearest Neighbor
EMD	Empirical Mode Decomposition
MFDCCA	Multifractal Detrended Cross-Correlation Analysis
CEEMD	Complementary Ensemble Empirical Mode Decomposition
ML	Machine Learning
RUL	Remaining Useful Life
SCMGIDTL	Spatial-Channel Collaborative Multi-Scale Graph Interaction Deep Transfer Learning
VIPINN	Machine-Vision-Driven, Physics-Informed Neural Network
IoT	Internet of Things
DTs	Decision Trees
IFD	Intelligent Fault Diagnosis
AD	Anomaly Detection
BDC	Bottom Dead Center
FFT	Fast Fourier Transform
GMF	Gear Mesh Frequency
LR	Logistic Regression
PLC	Programmable Logic Controller
